# Underestimation of Species Richness in Neotropical Frogs Revealed by mtDNA Analyses

**DOI:** 10.1371/journal.pone.0001109

**Published:** 2007-10-31

**Authors:** Antoine Fouquet, André Gilles, Miguel Vences, Christian Marty, Michel Blanc, Neil J. Gemmell

**Affiliations:** 1 Molecular Ecology Laboratory, School of Biological Sciences, University of Canterbury, Christchurch, New Zealand; 2 EA 3781 EGEE Evolution Genome Environment, Université de Provence, Centre St Charles, Marseille, France; 3 Division of Evolutionary Biology, Zoological Institute, Technical University of Braunschweig, Braunschweig, Germany; 4 Impasse Jean Galot, Montjoly, French Guiana; 5 Pointe Maripa, RN2/PK35, Roura, French Guiana; Ecole Normale Supérieure de Lyon, France

## Abstract

**Background:**

Amphibians are rapidly vanishing. At the same time, it is most likely that the number of amphibian species is highly underestimated. Recent DNA barcoding work has attempted to define a threshold between intra- and inter-specific genetic distances to help identify candidate species. In groups with high extinction rates and poorly known species boundaries, like amphibians, such tools may provide a way to rapidly evaluate species richness.

**Methodology:**

Here we analyse published and new 16S rDNA sequences from 60 frog species of Amazonia-Guianas to obtain a minimum estimate of the number of undescribed species in this region. We combined isolation by distance, phylogenetic analyses, and comparison of molecular distances to evaluate threshold values for the identification of candidate species among these frogs.

**Principal Findings:**

In most cases, geographically distant populations belong to genetically highly distinct lineages that could be considered as candidate new species. This was not universal among the taxa studied and thus widespread species of Neotropical frogs really do exist, contrary to previous assumptions. Moreover, the many instances of paraphyly and the wide overlap between distributions of inter- and intra-specific distances reinforce the hypothesis that many cryptic species remain to be described. In our data set, pairwise genetic distances below 0.02 are strongly correlated with geographical distances. This correlation remains statistically significant until genetic distance is 0.05, with no such relation thereafter. This suggests that for higher distances allopatric and sympatric cryptic species prevail. Based on our analyses, we propose a more inclusive pairwise genetic distance of 0.03 between taxa to target lineages that could correspond to candidate species.

**Conclusions:**

Using this approach, we identify 129 candidate species, two-fold greater than the 60 species included in the current study. This leads to estimates of around 170 to 460 frog taxa unrecognized in Amazonia-Guianas.

**Significance:**

As a consequence the global amphibian decline detected especially in the Neotropics may be worse than realised.

## Introduction

Amphibians are undergoing a drastic global decline [1]–[7]. Paradoxically, the number of amphibian species known to science is increasing with many new species discovered annually [8]–[11]. These new species descriptions are not the result of changes in theoretical species concepts but rather are a consequence of (1) real first hand discoveries (e.g. phenotypically divergent taxa described using traditional taxonomic practices), particularly due to the exploration of previously poorly known tropical areas [11], (2) diagnoses aided by molecular tools, and (3) the recent appreciation that a combination of slight differences in morphology and ecology (e.g. vocalisation) can be sufficient to characterize new species of amphibians [12] under both evolutionary and biological species concepts. However, despite these advances, to describe amphibian diversity and evolutionary history remains a difficult task because their morphological evolution is extremely conserved [13]–[16] and plagued with homoplasy [17]–[19]. Consequently, it is probable that a great proportion of amphibian diversity still remains to be discovered, not only at the species level but also in deeply rooted lineages, and this may be true for many other animal groups as well [20].

The Neotropics shelter the highest number of frog species on earth [21], [22], and this is also one of the region where amphibians are most threatened [7]. Many Neotropical frog species are thought to be distributed throughout Amazonia and adjacent areas [21], [23]. For example, although the Guianas are considered a single biogeographical entity due to the relative high endemism observed in the region, more than half of the currently recognised frog species in the Guianas occur elsewhere in Amazonia [21]. However, the idea that so many species have a widespread distribution is at odds with the low vagility and high philopatry observed in most amphibian taxa, characteristics that should promote differentiation and ultimately speciation [24]–[29]. Moreover, the view that so many species have a widespread distribution in the Neotropics conflicts with known historic climatic oscillations and geological events that have likely shaped the ranges of these Neotropical species and their ancestors [30]–[37]. This led Lynch [38] and Wynn and Heyer [39] to question respectively how many widespread frog species really exist, or if they indeed exist at all.

To decipher and fully understand amphibian diversification, an acceleration of comprehensive systematic revisions integrating morphological, bioacoustic and genetic data is needed. However, if the underestimation of species richness in Neotropical frogs observed in many groups by many authors [40]–[43] is ubiquitous the conservation implications for this threatened group are severe. Thus, there is an urgent need for an approach that can be used to rapidly obtain minimum estimates of the number of undescribed species in this group, and thereby identify priorities for taxonomic research and conservation actions. It has been argued that DNA sequences provide such a tool [44]–[47], and for the purpose of taxonomy, they can be analysed using three complementary approaches: phylogenetic analysis, comparison of molecular distances, and inferences from isolation-by-distance (IBD) calculations.

Phylogenetic analysis of DNA sequences can lead to the recognition of paraphyletic or polyphyletic gene lineages within *a priori* species. For mitochondrial DNA, species polyphyly and paraphyly have been found to be taxonomically widespread and far more common than generally recognized [48]. Such heterophyletic species designations are, in most cases, indeed indicative of incomplete taxonomy, which is when species names fail to identify the genetic limits of separate evolutionary entities [48]. Hence, the prevalence of species paraphyly or polyphyly can be used as an indicator for the number of yet undescribed species in a lineage. However, the reliability of the method is obscured by the possibility of incomplete lineage sorting, and by introgression that can cause gene heterophyly, especially in mitochondrial genes [49].

Another approach that can provide information on polyphyletic species is based on sequence divergences and thresholds for these distances. Vences et al. [46], [47] suggested that distance-based DNA barcoding could be a useful tool for documenting amphibian biodiversity. Pairwise divergences among sequences are calculated, and if these are above a previously defined threshold, the two sequences potentially belong to different species. If one of the sequences differs from all known species by a divergence above the threshold, it can be flagged as a “candidate species” and subjected to detailed taxonomic study [46]. However, because species-formation is a continuous process and the distinctive key characters (e.g., factors for prezygotic or postzygotic isolation) can evolve either early or late in this process [50], there necessarily are a number of very young (and hence genetically poorly differentiated) species that will be missed by the threshold-based estimates (false negatives). Again, because of introgression or incomplete lineage sorting, quite divergent lineages may not represent different species (false positives) [51]. Despite these pitfalls, a few studies on the distribution of the genetic diversity using mitochondrial DNA in different groups have shown that a gap exists between intraspecific and interspecific genetic diversity in some taxonomic groups. This gap is very clear in North American birds [45] and limited overlap has been found in Chironomidae (Diptera) [52], in climbing salamanders (*Aneides*), mantellid frogs [46] and cowries [51]. Threshold values therefore should be set high enough to ignore, as much as possible, intraspecific divergence, but low enough to ensure detection of as many incipient or newly emergent species as possible. In amphibians, thresholds of 0.05 ( = 5%) for a fragment of the 16S rRNA gene and of 0.1 ( = 10%) for the Cox1 gene have been proposed [46], [47].

In a group with low vagility like frogs, the main factor supposedly driving genetic differentiation among conspecific populations is isolation by distance (IBD) [53]. Moreover, the most common mode of amphibian species formation is supposed to be allopatric speciation [54]. In this scenario, a strong correlation between genetic and geographic distances is expected among populations of the same species [53]. However, once (allopatric) speciation is completed, secondary contact and overlap among the ranges of sister species is to be expected, decreasing the correlation between genetic and geographical distances [55], [45]. Hence, as long as distances between related populations follow an IBD model they can be considered, with some probability, to be conspecific. In contrast, where differentiation cannot be explained by simple IBD models, it is likely that more than one species is involved.

Here, we use a combination of published and new 16S mitochondrial rDNA sequences from 60 frog species known to occur in French Guiana, most of which are considered to be widely distributed across the Guianan and Amazonian regions, to obtain a minimum estimate of the number of undescribed species of amphibians in this region. We base our analyses on the three methods described above, and furthermore combine the IBD and distance-based analysis to evaluate threshold values for the identification of candidate species in amphibians.

## Results

### Prevalence of paraphyletic species

**Figure 1:**
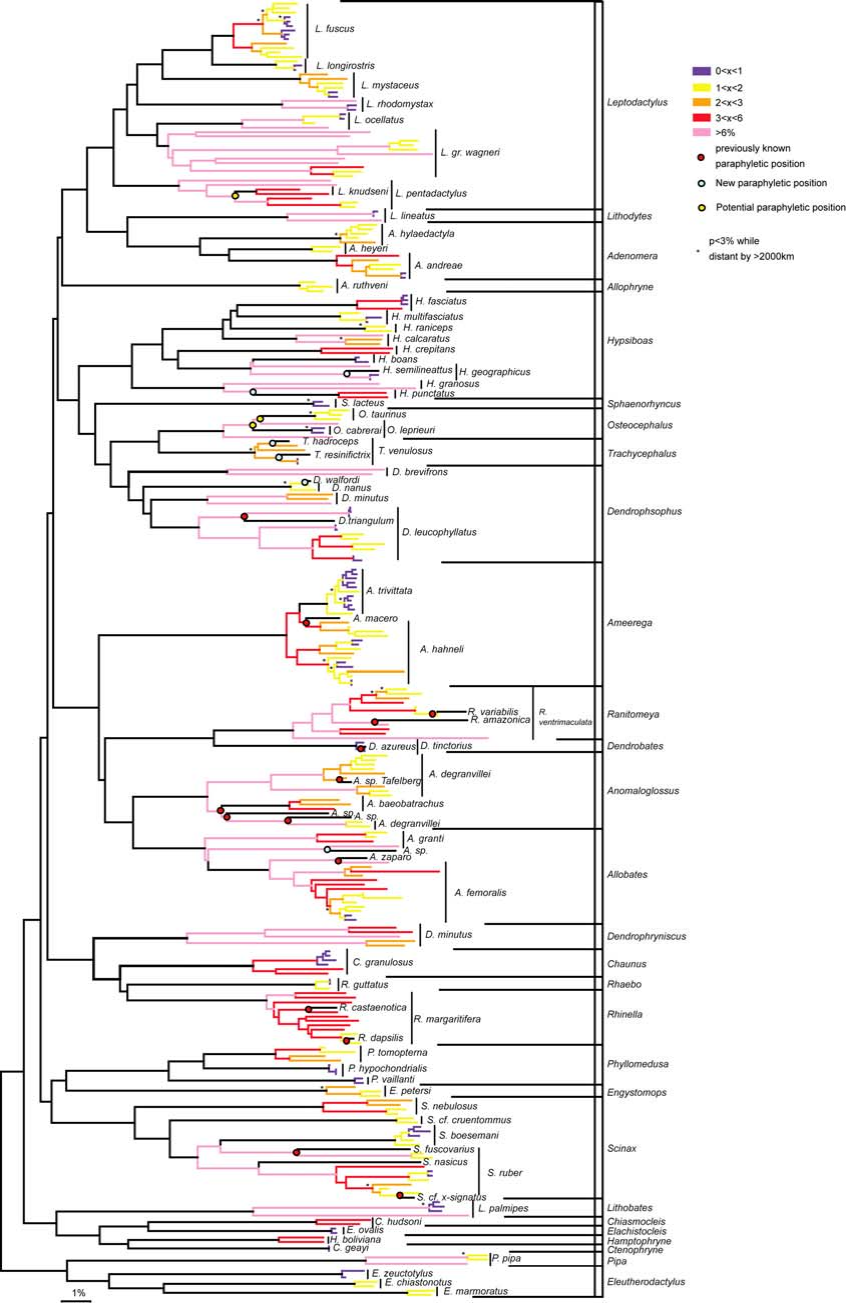
A Neighbour-Joining phylogram using p distances among 285 sequences representing 60+18 species. Branches are coloured in blue for intraspecific distances between 0 and 1%, in yellow for distances between 1 and 2%, in orange for distances between 2 and 3%, in red for distances between 3 and 6% and in pink for distances higher than 6%. Circles represent paraphyletic position either revealed by previous study (Red) or in the present study (blue) supported by high (>75) bootstrap values (ML and MP) and posterior probabilities, Yellow circles when the relationship between the species is not resolved and potentially paraphyletic. Asterisks represent close lineages (<3%) which occur at localities more distant than 2000 km.

DNA sequences were available for only a fraction of taxa potentially related to our target species. Nevertheless we found 13 out of our 60 target species (22%) displaying strongly supported paraphyletic relationships according to the Bayesian analyses (Figure 1, Figure S2, Figure S3). Eight of these had been previously recognized, for example, *Scinax ruber* with respect to *S. fuscovarius* and to *S. x-signatus* [56] and *Dendropsophus leucophyllatus* with respect to *D. triangulum* [40] and seven were novel. Ten of these 13 species have at least one lineage closer to another species than to the other conspecific lineages, with distances below 0.06 between them. The remaining species (outside the 13 above) formed strongly supported monophyletic groups except three ambiguous cases with low posterior probability: *Leptodactylus fuscus* (*L. longirostris* nested within), *Osteocephalus leprieurii* (*O. cabrerai* and *O. taurinus* nested within) and *Leptodactylus pentadactylus* (*L. knudseni* nested within).

### Patterns of intraspecific distances

**Figure 2:**
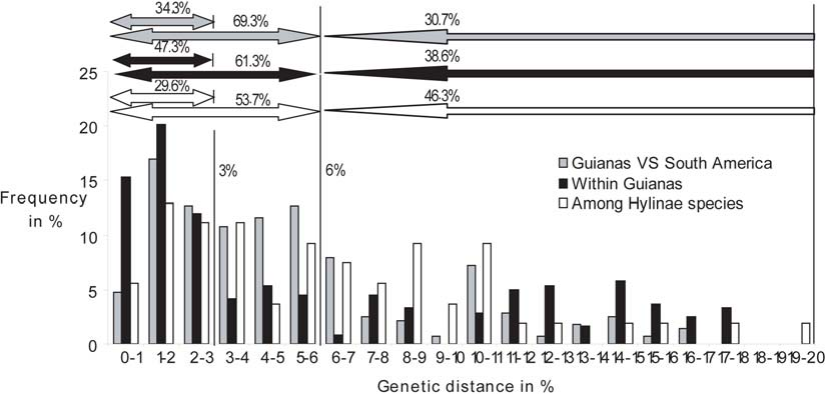
A histogram showing the distribution of the pairwise genetic distances among (1) conspecific populations from the Guianas versus other populations in South America (Grey), (2) conspecific populations within Guianas (Black), (3) closest Hylinae species from the dataset of Faivovich et al. (2005) (White). The arrows above the histogram provide summary data showing the proportion of distances in each of the three categories situated between 0 and 3%, 0 and 6% and above 6%.

Twenty-one out of 60 species (35%) contain lineages that differ from each other by uncorrected distances over 0.06, and 35 species (58.3%) contain lineages differing by more than 0.03 (Figure 2). The 0.06 limit segregates 94 lineages instead of the 60 species (56.7% more) included in this study and the 0.03 limit segregates 129 lineages (115% more).

Despite having been sampled in very distant localities (more than 2000 km), sixteen species display close lineages (less distant than 0.03) and four display very close lineages (less than 0.01) (Figure 1, Figure 2). For example, *Dendropsophus nanus* lineages from French Guiana and Argentina have a divergence of only 0.014 but are more than 3200 km apart (Figure S1). However, our pattern fits with geography in certain aspects. Half (46.7%) of the pairwise distances among Guianan populations were between 0 and 0.03 while only one third (35.4%) of the comparisons between Guianan and other South American populations were under 0.03 (Figure 2). The very low divergences, considered here as distances within a lineage (between 0 and 0.01), are much more frequent among Guianan populations (15%) than between Guianan and other South American populations (5%) (P(Chi^2^) = 4.8×10^−5^, ddl = 1, N = 520). Conversely, distances between 0.03 and 0.06 are found in 14.2% of the among-Guiana comparisons and 35.4% of comparisons between Guianan and other South American populations (P(Chi^2^) = 3.6×10^−8^, ddl = 1, N = 520). Indeed, distances below 0.03 are significantly more common among Guianan lineages than between lineages from South American and Guiana (P(Chi^2^) = 0.002, ddl = 1, N = 520).

In contrast, the proportions of very high distances (>0.06) among conspecific populations are only slightly different between populations within Guianas (39.2%) and Guianas vs. South America (29.3%) (Figure 2). Such incongruence between geography and genetic patterns can also be seen in Figure 1. In 14 species in which at least two lineages occur in French Guiana, one of them is closer to a lineage occurring elsewhere in South America (*H. fasciatus, H multifasciatus, H. geographicus, R. ventrimaculata, A. degranvillei, A. granti, S. ruber, S. boesemani*, *R. margaritifera, L. longirostris, L. mystaceus, A. andreae, A. hylaedactyla, L. gr. wagneri*). Reciprocally, in 12 species (*L. fuscus, L. pentadactylus, L. palmipes, H. calcaratus, A. hahneli,* *A. trivittatus, R. ventrimaculata, A. femoralis, C. granulosus, R. margaritifera, S. ruber, P. pipa*) one of the South American lineages is closer to one of the Guianan representatives than at least one other conspecific lineage in the rest of South America (Figure 1 appendix).

### Patterns of interspecific distances

The distribution of interspecific p distances using Hylinae widely overlaps with the intraspecific distributions (Figure 2). Indeed, the distribution of the genetic distances between Guianan versus South American populations and the distribution of interspecific pairwise distances are almost similar. More than half (53.7%, 29/54) of the interspecific distances correspond to values below 0.06. Still, 29.6% (16/54) of the apical distances correspond to values below 0.03.

### Isolation by distance

**Figure 3:**
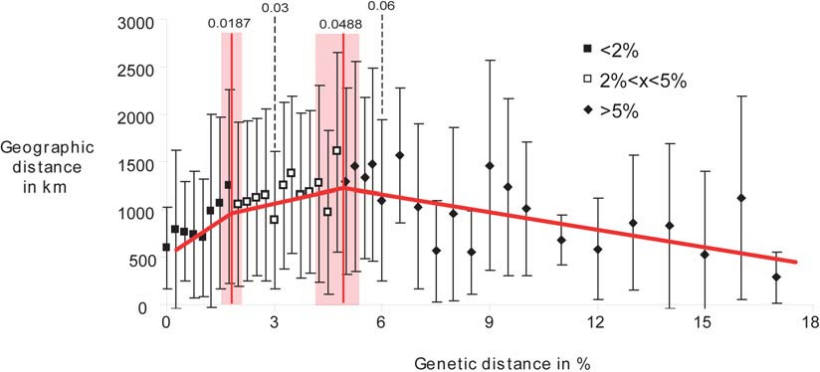
The distribution of the pairwise genetic distances among conspecific populations against geographical distances (N = 822). Genetic data are segregated by 0.025% classes from 0 to 6%, by 0.5% classes from 6 to 10% and then by 1% for higher values. Linear models computed from the distribution of the raw data.

According to the BIC, the selected model explaining the relation between geographical and genetic distances was made up of 3 linear models (Figure 3). The first one concerns genetic distances between 0 and 0.016 and has a strong positive slope (2.5×10^5^±0.29×10^5^). The second one concerns the genetic distances between 0.016 and 0.048 and has a three-fold weaker but still positive slope (9×10^3^±4.4×10^3^). Genetic distances that are over 0.048 are best fitted with a negative slope.

## Discussion

### Deep polyphyly and paraphyly suggest a high proportion of cryptic species

Our data indicate a high number of potentially new frog species occurring in the Guianan and Amazonian region. This conclusion is supported by (1) the high genetic divergences among lineages within species and (2) by the presence of many paraphyletic species. Depending on the method used, the proportion of candidate species relative to the 60 study species varies from 22–115%.

In Hylinae, most distances between sister species (53.5%) were below 0.06 and one third was even below 0.03. This indicates that divergences corresponding to intraspecific distances over 0.03 can be considered as deep. Indeed the intraspecific and interspecific distances distributions widely overlap. While 53.5% of the interspecific data were below 0.06, this was the case for 61.3–69.3% of the intraspecific data (Figure 2). The number of deeply related intraspecific lineages is very high: 94 lineages are more distant than 0.06 and 129 lineages are more distant than 0.03, giving proportions of 56% and 115% of candidate new species.

The phylogenetic analysis demonstrated paraphyly of lineages within 13 species out of 60. Hence, this approach suggests in 22% of cases current species designations do not adequately represent true species designations.. This, is a maximum estimate given the data, because in some cases it may represent introgression through recent hybridization, incomplete lineage sorting, or erroneous phylogenetic reconstruction. On the other hand, few species of Neotropical amphibians have been sequenced for this mtDNA fragment so far [57], and thus the potential of the available data to detect paraphyly is small, suggesting that this situation might be much more frequent than it is shown by the data herein. This phenomenon is taxonomically widespread and also corroborated by recent studies for other groups of frogs (e.g. *Pseudae* [58]; *Chaunus marinus* [59], Central American *Brachycephalidae* [60] and in other parts of the world [61], [62]. In Malagasy mantellids and North American salamanders, the overlap between intra- and inter-specific distances is smaller and allows of setting more clearly a threshold values. We assume that this is because their systematics have been extensively studied and their taxonomy is now better fitting their respective evolutionary histories than is the case for most Neotropical frogs. Indeed, the taxonomic coverage of DNA sequence data is one the highest for Malagasy frogs and North American Caudata while it is one of the lowest for Neotropical frogs [57].

### Widespread species of Neotropical frogs do exist

Our analysis suggests that widespread Neotropical frog species do exist [38], [39]. Here we have confirmation that conspecific populations (*Osteocephalus cabrerai*, *Osteocephalus taurinus, Sphaenorhynchus lacteus*, *Lithobates palmipes*, *Pipa pipa, Hypsiboas boans*) are genetically so close that they probably belong to one widespread species which has dispersed over vast areas in South America (Figure S2). Nevertheless, it seems that widespread lineages are a minority (in our dataset 16 out of 53; 60 species considered in total less seven species purportedly endemic to the Guianan shield). However, species can be at the same time widely distributed and contain candidate new species: in *Pipa pipa*, even if one lineage was widely distributed, the species was still found to be deeply polyphyletic. Low sampling might mask a similar pattern in other species, and further work to determine this is warranted. It is worth mentioning that most of these widespread species are associated with open areas (*Leptodactylus fuscus*, *Adenomera hylaedactyla*, *Scinax ruber*) or with rivers or large swamps (*Lithobates palmipes*, *Pipa pipa*, *Sphaenorhynchus lacteus*, *Hypsiboas raniceps*, *Dendropsophus nanus*).

### Geographical data also support the idea that deep lineages may be considered as candidate new species

The comparison between genetic and geographical distances (Figure 3) seems to fit the expectations about the process of speciation by allopatry. The strong association between geographical and genetic distances between 0 and 0.019 is certainly due to intraspecific variation among populations mainly driven by isolation by distance. The absence of strong correlation between genetic and geographical distances for distance values over 0.019 is probably due to the increase of the number of allopatric species displaying no contact or superficial contact/hybrid zones, and sympatric species [56]. The data over 0.049 probably include a prevalence of sympatric species that are likely to be reproductively isolated from each other [56].

Moreover, a series of discordant relationships between geography and genetic distances can be detected: (1) in many species, one of the lineages detected within French Guiana is closer to a population sampled elsewhere in South America; (2) the distribution of the pattern of distances on a small geographical scale (within Guianas) and a large one (between Guianas and South America) is basically the same, suggesting that these lineages could represent different species in contact in French Guiana; (3) in *Scinax ruber*, *Rhinella margaritifera* [56], *Leptodactylus* gr. *wagneri*, *Anomaloglossus degranvillei*, *Allobates femoralis* [41], [63], *Dendropsophus leucophyllatus* [40], [63], *Ameerega hahneli* and *Ameerega trivittata* [41], [43], for example, the distributions of some lineages and their relationships are clearly discordant and suggests that some of these lineages could be sympatric (Figure 1 in appendix).

### A divergence threshold value of 0.03 to identify amphibian candidate species

Based on the isolation by distance analysis, a threshold between 0.019 and 0.049 appears to be appropriate to distinguish between intraspecific and interspecific divergences among Neotropical anurans. Several additional lines of evidence support a threshold around 0.03:

1. Divergences within vs. among regions: In Figure 2 (see also the Chi^2^ analyses), the distances calculated among Guianan populations mainly range between 0 and 0.03 whereas the comparisons between Guianan and other South American populations predominantly yielded distance values between 0.03 and 0.07. This also can be interpreted as a dominance of intraspecific distances mainly driven by isolation by distance between 0 and 0.03 and over that threshold, the predominance of pairwise distances between allopatric species distributed in the Guianas and in other regions of South America, respectively.

2. Concordance with assumed ages of speciation. The genetically and geographically highly distant conspecific populations have probably been isolated during the recent geological period of climatic oscillations and geological events and many of them have probably remained isolated since this time. The majority of recent speciation events for amphibians seem to have occurred before the Pleistocene period [40], [63]. This pattern is also observed in birds, primates and rodents in South America [64]–[67]. A calibration of 0.0037 to 0.006 divergence per million years for tRNA, and 16S rDNA by Evans et al. [68] predicts a divergence of 0.0066 to 0.011 on 16S rDNA between closely related species that last share a common ancestor dating from the Plio-pleistocene limit (1.8My bp) (similar proportions of substitutions are observed with the mtDNA fragment used by Evans et al. and our smaller fragment size). Assuming many lineages emerged at the Plio-Pleistocene period, this would again suggest that the 0.03 threshold is a more reasonable predictor of lineages describing potential candidate species than the 0.06 threshold.

3. Concordance with well-sampled datasets. The 0.03 threshold segregates 70% (versus 46% false negative with 0.06) of the terminal divergences in the dataset of Faivovich et al. [69]. Moreover, some of the species below the 0.03 threshold might actually deserve to be synonymised (false negatives) as it has been the case recently for *Dendrobates azureus* and *D. tinctorius* [70]. Fouquet et al. [56] delimited *Scinax ruber* and *Rhinella margaritifera,* lineages that that correspond to reproductively isolated species with divergences as low as 0.0385 (*R. margaritifera* A versus D). They also found five further lineages of lower divergences that may represent distinct species as well given the positions of *Scinax x-signatus* and *Rhinella dapsilis* which are nested among the lineages with low genetic distances. The pattern obtained for the interspecific distances using the dataset of Faivovich et al. [69] data overestimates genetic distances between sister species because distances used are not only between sister species but concern deeper relationships as well. The Hylinae clade is not sampled with sufficient rigour to solely examine distances between sister species. It is therefore likely that some high distances observed are actually between distantly related taxa.

These arguments advocate the use of a 0.03 (3%) threshold to identify candidate species of Neotropical anurans and reject the adequacy of the 0.06 (6%) threshold proposed previously. The 0.3 (3%) threshold is preferred to either higher of lower thresholds because a higher threshold (e.g. 0.06) risks missing many potential species while a lower threshold (e.g. 0.02) will more accurately delimit lineages but risks identifying many conspecific lineages as candidate species.

Genetic diversity has been demonstrated to be higher within tropical species than in the temperate species [71]–[73]. Indeed, the trend for population differentiation to increase with decreasing latitude was used by Moritz and Cicero [74] to argue against the broad application of such a DNA distance based metric for delineating biodiversity in the tropics. While we did not observe a strong disjunction between the intraspecific and the interspecific pairwise distance distributions in tropical frogs in our data set, Vences et al. [46], [47] did observe such a gap. Moreover, the levels of divergence between lineages, populations and even most sister species in temperate areas reside well below the 3% threshold in sequence difference we suggest for 16SrDNA in this study [75]–[77]. Consequently, we believe that a 3% threshold may prove to be a useful tool to document tropical frog biodiversity in a wide variety of contexts.

### Conclusions

Our results clearly show that the number of species is highly underestimated in anurans from the Guianan and Amazonian regions. Our approach indicated that up to 115% additional species may be expected among Neotropical amphibians. About 400 anuran species are currently recognised in Amazonia-Guianas, with 37% of these species (about 150) having ranges >1 million km^2^ that can be considered as sufficiently widespread for an extrapolation of the number of potential cryptic species. Extrapolating from our data, the total number of species in this region might easily approach 600 (400-150+(150*215%)). However, even if our analysis comprises the most widespread species inhabiting Amazonia-Guianas (85% of the species included have ranges >1 million km^2^) this extrapolation is likely to be a minimum estimate. Two reasons may account for this: (1) given the low proportion of most of the ranges sampled in our analysis many more extant lineages may have remained unsampled; (2) many species that are currently considered of restricted range are poorly known and their ranges might be wider. If we apply this extrapolation to the total number of species in Amazonia this would lead to a total number of over 860 (400*215%) and over 4400 (2065*215%) for South America [12]. Of course these estimates are extremely rough, but even the lowest estimate of 22% new species (considering only the paraphyly criterion) leads to almost 490 (400*122%) species for Amazonia-Guianas and almost 2520 (2065*122%) for South America that are to be expected without considering true first-hand discovering which also are going on at a fast pace.

Species delimitation is essential for conservation of biodiversity, especially in the tropics where indicators such as the species richness or the degree of endemism are simple and efficient indicators of biodiversity that can be monitored for change over time. To be accurate, the species delineation needs to use a taxon specific approach (by genus or group of species) using a combination of data from phylogenetic, phylogeographic, morphological and ecological data [78]. However, considering the enormous number of new candidate species detected by our analysis it is clear that such analyses would take considerable time. However, biodiversity data are urgently needed to help define conservation priorities. Molecular diversity data may be useful surrogates for evaluate amphibian biodiversity before it vanishes. Even if some of the lineages identified may ultimately be shown not to represent species, while others may be missed, the net gain in amphibian diversity in regions like the Neotropics makes such a strategy attractive.

As a consequence of the underestimation of the number of frog species, the global amphibian decline detected especially in the Neotropics may be worse than so far realised [11], [7]. Indeed, we cannot know how many “species” instead of “populations” have already disappeared or are disappearing, and the situation is particularly acute in the tropics. The rapid identification and recognition of new species may exacerbate an organism's threat status because it can result in the subdivision of a once widespread species into numerous species, each with a smaller and, hence, a more precarious distribution. Nevertheless, it is obviously better to know the state of biodiversity threat than to be ignorant of the mammoth changes in global amphibian diversity that we are witnessing.

## Materials and Methods

(Further details about the methods used are available in Text S1.)

### Sequences and laboratory protocols

We selected available sequences in GenBank attributed to 60 of the 102 anuran species (28 genera) known to occur in French Guiana (445 sequences) according to Boistel et al. [79] and Lescure and Marty [80]. To this, we added sequence data from 69 individuals sampled in French Guiana and 25 individuals sampled elsewhere in South America (Table S1). Each sequence was attributed to one of 60 currently designated species (two to 38 sequences per species; Table S1), most of which (88.7%) are currently considered to be widespread across the Guianan and Amazonian regions (see supplementary materials).

DNA was extracted using either standard phenol chloroform or lithium chloride methods [81]. Primers used for amplification are described by Salducci et al. [82] for 16S rDNA. PCRs were performed in a 25-µl total volume with cycle parameters as described in Salducci et al. [82]. Sequencing was performed using ABI Big Dye V3.1 and resolved on an automated sequencer at Macrogene Inc. (Korea) and the University of Canterbury sequencing service (New Zealand).

Preliminary alignment of sequences was performed with Clustal X [83] with a gap penalty equal to five, with other parameters set at the default settings. Each alignment was verified by eye and compared with secondary structures (16S rDNA) [84]. Newly determined sequences were deposited in GenBank (Table S1).

The final alignment of the 16S rDNA fragment was 420 base pairs, a slightly shorter fragment than that used by Vences et al. [46], but containing a high proportion of the polymorphic sites detected in this gene segment. Comparing the pairwise distances of the two fragment sizes employed by this study and the earlier work of Vences et al. [46] results in a ratio of 1.2 (R^2^ = 0.99; p = 0.0001, df = 52) (Figure S1). Thus, the 5% divergence threshold proposed by Vences et al. [46] corresponds to a 6% threshold with our fragment size.

We chose to use this fragment for several reasons: (1) It is the most commonly used marker for amphibian systematics and thus the DNA fragment for which the taxonomic sampling is currently the highest [46]. (2) It is easy to obtain for a wide array of groups because of highly conserved region (hairpins) flanking more variable region (loops) and also for other reasons detailed by Vences et al [46], [47]. Some authors, arguing against the use of this gene, have suggested that sequence alignment can be problematic due to indels occurring within the highly variable loop regions. This indeed is often the case for deep relationships and it is well known that coding mtDNA such as *cox1* displays some advantage due to the conservation of the reading frame which usually provides unambiguous guidance for a global alignment [85]. However, because our analyses only deal with closely related taxa the alignment is unambiguous and the advantage of the large sequence set available for 16S far outweighs those of easier alignment of the more limited *cox1* data.

### Assessment of species monophyly

For each of the species studied we selected 16S sequences as “lineages” that had higher uncorrected pairwise distances than 0.01 ( = 1%) from the closest other sequence in the analysis. Previous work in two groups of frogs (*Scinax ruber* and *Rhinella margaritifera*) included in this study showed that intraspecific diversity clusters into haplogroups for which the diversity is circumscribed between 0 and 0.01 [56]. Multiple representatives of lineages, which we called “populations”, were selected only when they occurred at several remote localities (i.e., different states or countries).

To test the monophyly of each species we first selected, from GenBank, all available sequences attributed to putatively closely related species that potentially could nest among the identified lineages of any of our study species. To select these additional species, we (1) selected taxa which displayed a close relationship with the species studied according to previous work (see references and additional details in supplementary materials) and (2) using the BLAST option with all the previously selected sequences of the species studied. We chose the first hit of a heterospecific sequence in each case.

Subsequently, preliminary phylogenetic analyses were performed for each species using Maximum Parsimony implemented in PAUP 4.0 [86]. Confidence in the phylogenetic grouping was assessed by the non parametric bootstrap method [87], [88] with 1000 pseudoreplicates undertaken using the heuristic search option, tree bisection reconnection branch swapping (TBR) and 10 random taxon addition replicates. For each analysis we used all the sequences from conspecific populations, the alternative heterospecific sequences potentially introducing paraphyly and a supposedly closest species as outgroup. Only the alternative species nesting with strong bootstrap support within already selected species were kept. Subsequently, a Bayesian phylogenetic analysis was performed with MrBayes 3.1 [89] on the complete dataset. We used the software Modeltest version 3.6 [90] to choose the substitution model that best fits our data using the Akaike Information Criterion [91]. These models (Text S1) were subsequently used for Bayesian analysis on the University of Canterbury Supercomputer. Bayesian analysis consisted of 2 independent runs of 1.0×10^7^ generations with random starting trees and four Markov chains (one cold) sampled every 1000 generations. Adequate burn-in (1.0×10^6^) was determined by examining a plot of the likelihood scores of the heated chain for convergence on stationarity. We flagged those nodes which received posterior probabilities >80 as probably supporting paraphyly.

### Comparisons of intraspecific distances

Sixty of the 102 currently known anuran species in French Guiana (59%, representing 28 of 36 genera) were used in the current study. These species were represented by 539 sequences, of which 221 lineages were identified after discarding 318 redundant sequences that corresponded to sequences belonging to already included lineages and originating from the same or very close localities as those already in the analysis (Table S1). We calculated 825 pairwise distances between conspecific lineages; of these, 240 distance values were between lineages sampled within the Guianas representing 43 species, and 246 between Guianan lineages and other South American lineages, representing 33 species.

Using the uncorrected pairwise distances, we constructed a neighbour joining tree using MEGA 4 [92]. We then plotted the distribution of these distances in two categories “Guianas against South America” and “within Guianas” to check whether the pattern differs between biogeographical regions.

We calculated how many lineages are separated by the 6% threshold, and repeated this analysis with a 3% threshold as lower limit based on data from Fouquet et al. [56] that provided evidence that reproductively isolated cryptic species can be separated by 3.8% (*Rhinella*) and 4.3% (*Scinax*) based on 16S rDNA sequences.

### Interspecific distance distribution: the example of Hylinae

To compare the distributions of intraspecific distances calculated above with a distribution of validated interspecific distances, we used homologous 16S rDNA fragments from the dataset published by Faivovich et al. [69] because of its very complete taxon coverage for a group of Neotropical frogs (Hylinae). From this dataset, we chose of species that were fully resolved as sister species in the original analysis [69] in order to capture the most recent speciation events. We eventually used 108 species (54 pairs) to compute the interspecific distance distribution.

### Isolation by distance and species range data

To test whether or not the critical levels of the intra- and inter-specific distance distributions that we determined *a priori* fit expectations about IBD, we plotted genetic distances against geographic distances (N = 822). A Mantel test is not applicable with these kinds of data, where pairs of intraspecific lineages are compared and pooled altogether. Thus, we described the relation between geographical distances and pairwise genetic distances using a piecewise linear model [93]. The parameters of the model were estimated by the least squares method. We used the Bayesian information criterion (BIC) to choose the adequate model (i.e. number of pieces, up to 6) that best fit the raw data. This procedure was implemented using R 2.5.0 (R Foundation for Statistical Computing, 2005) and was repeated 10 times with a random start. The best model was kept and 95% confidence intervals were estimated using 500 random resamples. Additionally, genetic distances between pairs were grouped into classes and the means and variance of geographical distances was calculated for each class (Figure 3).

Approximate range sizes of the anuran species occurring in Amazonia-Guianas were estimated from the Global Amphibian Assessment (GAA) database (www.globalamphibians.org). The delimitation of Amazonia followed the Amazonia wilderness area and only species occurring broadly in this area were selected. Subsequently, we removed species occurring fully or partially above 600m, in order to avoid including species restricted to the Andes and the Guiana highlands.

## Supporting Information

Text S1 Additional details on material and methods and additional references.
(0.05 MB DOC)

Table S1 Sample details and accession numbers. Names in grey correspond to additional species used in the figure to illustrate paraphyletic positions. X and O are used to indicate which sequences have been discarded from the analyses.
(0.08 MB PDF)

Figure S1 Distribution of the pairwise distances between the Hylinae sister species from Faivovich et al. (2005) with two sizes of the same 16S rDNA fragment: One with 590bp corresponding to the fragment used by Vences (2005) and one with 419bp for the present study.
(3.99 MB TIF)

Figure S2 Tree from Figure 1 with sample labels and geographical indications: FG = French Guiana; SUR = Suriname; GUY = Guyana; VEN = Venezuela; BR = Brazil; COL = Colombia; PAN = Panama; CR = Costa Rica; ECU = Ecuador; PER = Peru; BOL = Bolivia; PAR = Paraguay; ARG = Argentina.
(6.89 MB TIF)

Figure S3 Consensus tree derived from Bayesian analysis of the data
(2.31 MB TIF)
