# Supplementary material for: Underestimation of Species Richness in Neotropical Frogs Revealed by mtDNA Analyses
Source: PLoS One. 2007 Oct 31;2(10):e1109. doi: 10.1371/journal.pone.0001109 (PMC2040503; doi:10.1371/journal.pone.0001109)
Supplement: Text S1 — Additional details on material and methods and additional references. (0.05 MB DOC) [file pone.0001109.s001.doc]

**Text S1:**

Additional details about materials and methods:

1. Except for seven species purportedly endemic to the Guianas (*Dendrobates tinctorius*, *Anomaloglossus degranvillei*, *Anomaloglossus baeobatrachus*, *Allobates granti*, *Chiasmocleis hudsoni*, *Adenomera heyeri* and *Eleutherodactylus chiastonotus*), all the selected species are believed to be widely distributed across the Guianas and Amazonia (88.3%).

2. If 104 species are supposed to occur in French Guiana according to Lescure and Marty (2000) and Boistel et al. (2006), we only counted 102: Three species of the *Leptodactylus wagneri* species group (*L*. gr. *wagneri*) are supposed to occur in French Guiana (*L. leptodactyloïdes*, *L. petersi* and *L. pallidirostris*). We have been unable to accurately identify morphologically these species in French Guiana. There are no published sequences for these species except for *L. leptodactyloïdes*. Consequently, they are treated here as a single species. We are conscious that it biased the analyses but we think it was the most parsimonious way to deal with this group. Moreover, not to take this group into account would not have changed the results and their interpretation.

3. Heyer (2005) clarified the species boundaries in the *Leptodactylus pentadactylus* species group during the writing of the manuscript. Consequently the different *L. pentadactylus* lineages used herein represent different species than described in Heyer (2005).

Heyer, W. R. (2005). Variation and taxonomic clarification of the large species of the *Leptodactylus pentadactylus* species group (Amphibia: Leptodactylidae) from Middle America, Northern South America and Amazonia. *Arquivos de Zoologia do Estado de São Paulo* **37**(3), 269–348

4. One sequence attributed to *Adenomera andreae* published by Faivovich et al. (2005) unambiguously clustered in the *Leptodactylus wagneri* species group in preliminary phylogenetic analyses. This result is likely due to misidentification of the specimen. We called this taxon *Leptodactylus gr. wagneri* J.

5. We chose to keep the genera *Leptodactylus*, *Adenomera* and *Lithodytes* because we found arguments in Frost et al. (2006) for their synonymy to be ambiguous. Frost et al. (2006) based this recommendation on the work of Heyer (1998) in which the genus *Adenomera* is represented by only one species, its position, analysed with morphology and vocalisation, is not fully resolved and is not the main purpose of the article. Moreover, see comment 4 above.

Heyer WR (1998) The relationships of *Leptodactylus diedrus.* *Alytes* **16**(1-2): 1–24.

Recent works published during the writing of this manuscript also support taxonomic modifications for the genera *Eleutherodactylus* (Heinicke et al., 2007) and *Chaunus* (Pramuk et al., 2007).

Heinicke MP, Duellman WE, Hedges SB (2007) Major Caribbean and Central American frog faunas originated by ancient oceanic dispersal. *Proceedings of the National Academy of Sciences of the United States of America* **104**(24): 10092–10097.

Pramuk JB, Robertson T, Sites Jr JW, Noonan BP (2007) Around the world in 10 million years: biogeography of the nearly cosmopolitan true toads (Anura: Bufonidae). *Global Ecology and Biogeography* (in Press).

6. We also sampled and sequenced all other congeneric species supposedly occuring in French Guiana (23 species except *Chaunus marinus*, *Dendropsophus marmoratus*) according to Lescure and Marty (2000) for the same 16S fragment to avoid any misidentification of the specimens collected (data not shown, see appendix 1 supplementary material). As there were no additional intraspecific data available inside or outside French Guiana these species have not been taken into account for the intraspecific analyses.

7. Such a selection was conducted to represent the geographical range of the lineages and because, (1) an evaluation of the diversity existing within each lineage requires extensive sampling and was not the purpose of this work; (2) the number of pairwise distances below 0.01 would be highly correlated by the number of individuals included within each lineages thus these data would have biased the analyses.

8. To test the monophyly of each species we selected in GenBank available sequences of additional species that were potentially nesting within species. To select these additional species, we used available data for taxa which displayed close relationship with the previously selected species according to previous work and using the Blast option with all the previously selected sequences. We chose the first hit if this sequence had not been already selected as a conspecific sequence. With this method we probably missed available data producing paraphyletic positions within species but our goal was not to evaluate the frequency of this phenomenon because the taxonomic representation of the available sequences would not allow us to evaluate it anyway, but only to show its appreciable presence and to be able to evaluate at which distances it occurs.

The following references are only used in supplementary materials as sources for the sequences information.

*Austin JD, Lougheed SC, Tanner K, Chek AA, Bogart JP, Boag PT (2002) A molecular perspective on the evolutionary affinities of an enigmatic Neotropical frog, *Allophryne ruthveni*. *Zoological journal of the Linnean Society* **134**(3): 335–346.

*Balakrishnan R (2005) Species concepts, species boundaries and species identification: A view from the tropics. *Systematic Biology* **54**: 689–693.

*Biju SD, Bossuyt F (2003) New frog family from India reveals an ancient biogeographical link with the Seychelles. *Nature* **425**: 711–714.

*Camargo A, de Sá RO, Heyer WR (2006) Phylogenetic analyses of mtDNA sequences reveal three cryptic lineages in the widespread neotropical frog *Leptodactylus fuscus* (Schneider, 1799) (Anura, Leptodactylidae). *Biological Journal of the Linnean Society* **87**: 325–341.

*Clough M,Summers K (2000) Phylogenetic systematics and biogeography of the poison frogs: evidence from mitochondrial DNA sequences. *Biological Journal of the Linnean Society* **70**: 515–540.

*Darst CR, Cannatella DC (2004) Novel relationships among hyloid frogs inferred from 12S and 16S mitochondrial DNA sequences. *Molecular Phylogenetics and Evolution* **31**: 462–75.

*de Sá RO, Heyer WR, Camargo A (2005) Are *Leptodactylus didymus* and *L. mystaceus* phylogenetically sibling species (Amphibia, Anura, Leptodactylidae)? *In*: Ananjeva N, Tsinenko O, editors. Herpetologica Petropolitana-Proceedings of the 12th Ordinary General Meeting of the Societas Europaea Herpetologica, August 12-16, 2003, St. Petersburg, Russia. *Russian Journal of Herpetology* **12**(Supplement): 90–92.

*de Sá RO, Heyer WR., Camargo A (2005) A phylogenetic analysis of *Vanzolinius* Heyer, 1974 (Amphibia, Anura, Leptodactylidae): Taxonomic and life history implications. *Arquivos do Museu Nacional, Rio de Janeiro* **63**: 707–726.

*Faivovich J, Garcia PCA, Ananias F, Lanari L, Basso NG, Wheeler WC (2004) A molecular perspective on the phylogeny of the Hyla pulchella species group (Anura, Hylidae). *Molecular Phylogenetics and Evolution* **32**: 938–950.

*Frost DR, Grant T, Faivovich J, Bain RH, Haas A, Haddad CFB, de Sá RO, Channing A, Wilkinson M, Donnellan SC, Raxworthy CJ, Campbell JA, Blotto BL, Moler P, Drewes RC, Nussbaum RA, Lynch JD, Green DM, Wheeler WC (2006) The amphibian tree of life. *Bulletin of the American Museum of Natural History* **297**: pp.370.

*Heyer WR, de Sá RO Rettig A (2005) Sibling species, advertisement calls, and reproductive isolation in frogs of the *Leptodactylus pentadactylus* species cluster (Amphibia, Leptodactylidae). *In*: Ananjeva N, Tsinenko O, editors. Herpetologica Petropolitana-Proceedings of the 12th Ordinary General Meeting of the Societas Europaea Herpetologica, August 12-16, 2003, St. Petersburg, Russia. *Russian Journal of Herpetology* **12**(Supplement): 35–39.

*Hillis DM Wilcox TP (2005) Phylogeny of the New World true frogs (*Rana*). *Molecular Phylogenetics and Evolution* **34**: 299–314.

*Hull DL (1977) The ontological status of species as evolutionary units. *In*: Butts R, Hintikka J, editors. Foundational problems in the special sciences, D. Reidel Publishing Company, pp. 91–102.

*Lougheed SC, Gascon C, Jones DA, Bogart JP, Boag PT (1999) Ridges and rivers: a test of competing hypotheses of Amazonian diversification using a dart-poison frog (*Epipedobates femoralis*). *Proceedings of the Royal Society of London Series B-Biological Sciences* **266**: 1829–1835.

*Loetters S Vences M (2000) Bemerkungen zur Nomenklatur und Taxonomie peruanischer Pfeilgiftfrösche. *Salamandra* **36**: 247–260.

*Pauly GB, Hillis DM Cannatella DC (2004) The history of a Nearctic colonization: molecular phylogenetics and biogeography of the Nearctic toads (*Bufo*). *Evolution* **58**: 2517–35.

*Pramuk JB (2006) Phylogeny of South American *Bufo* (Anura: Bufonidae) inferred from combined evidence. *Zoological Journal of the Linnean Society* **146**: 407–452.

*Pramuk JB, Hass CA Hedges SB (2001) Molecular phylogeny and biogeography of West Indian toads (Anura: Bufonidae). *Molecular Phylogenetics and Evolution* **20**: 294–301.

*Roberts JL, Brown JL, Schutle R, Wilfredo A, Summers K (2006) Rapid diversification of colouration among populations of a poison frog isolated on sky peninsulas in the central cordilleras of Peru. *Journal of Biogeography* **34**(3): 417–426.

*Roelants K Bossuyt F (2005) Archaeobatrachian paraphyly and pangaean diversification of crown-group frogs. *Systematic Biology* **54**: 111–126.

*Ron SR, Santos JC, Cannatella DC (2006) Phylogeny of the tungara frog genus *Engystomops* (=*Physalaemus pustulosus* species group; Anura: Leptodactylidae). *Molecular Phylogenetics and Evolution* **39**: 392–403.

*Ruvinsky I, Maxson LR (1996) Phylogenetic relationships among bufonid frogs (Anura: Neobatrachia) inferred from mitochondrial DNA sequences. *Molecular Phylogenetics and Evolution* **5**: 533–547.

*Salducci M-D, Marty C, Chappaz R, Gilles A (2002) Molecular phylogeny of French Guiana Hylinae: implications for the systematic and biodiversity of the Neotropical frogs. *Comptes Rendus Biologies* **325**(2): 141–153.

*Santos JC, Coloma LA, Cannatella DC (2003) Multiple, recurring origins of aposematism and diet specialization in poison frogs. *Proceedings of the National Academy of Sciences of the United States of America* **100**(22): 12792–12797.

*Summers K, Clough ME (2001) The evolution of coloration and toxicity in the poison frog family (Dendrobatidae). Proceedings of the National Academy of Sciences of the United States of America 98, 6227–6232.

*Summers K, Symula R, Schulte R (2001) Molecular phylogenetic evidence for a mimetic radiation in Peruvian poison frogs supports a Mullerian mimicry hypothesis. *Proceedings of the Royal Society B-Biological Sciences* **268**(1484): 2415–2421.

*Summers K, Weigt LA, Boag P, Bermingham E (1999) The evolution of female parental care in poison frogs of the genus *Dendrobates*: Evidence from mitochondrial DNA sequences. *Herpetologica* **55**: 254–270.

*Symula, R, Schulte R, Summers K (2003) Molecular systematics and phylogeography of Amazonian poison frogs of the genus *Dendrobates*. *Molecular Phylogenetics and Evolution* **26**: 452–475.

*Vences M, Kosuch J, Boistel R, Haddad CFB, La Marca E, Lotters S, Veith M (2003). Convergent evolution of aposematic coloration in Neotropical poison frogs: a molecular phylogenetic perspective. *Organisms Diversity and Evolution* **3**: 215–226.
