# Supplementary material for: Underestimation of Species Richness in Neotropical Frogs Revealed by mtDNA Analyses
Source: PLoS One. 2007 Oct 31;2(10):e1109. doi: 10.1371/journal.pone.0001109 (PMC2040503; doi:10.1371/journal.pone.0001109)
Supplement: Table S1 — Sample details and accession numbers. Names in grey correspond to additional species used in the figure to illustrate paraphyletic positions. X and O are used to indicate which sequences have been discarded from the analyses. (0.08 MB PDF) [file pone.0001109.s002.pdf]

| Species/lineage | voucher number | Accession number 16S | | Countries | Locality | References | Coordinates |
|---|---|---|---|---|---|---|---|
| *Adenomera andreae A* | 32mc | EU201044 | X | French Guiana | Montjoly | New | 04 55 00 N 52 16 00 W |
| *Adenomera andreae B* | 216mc | EU201046 | X | French Guiana | Saül | New | 03 37 32 N 53 12 26 W |
| *Adenomera andreae B* | 105AF | EU201047 | X | Suriname | Road to Apura | New | 05 11 00 N 55 39 00 W |
| *Adenomera andreae C* | 87bm | EU201048 | X | French Guiana | Mt Arawa | New | 02 48 59 N 53 21 59 W |
| *Adenomera andreae D* | 199bm | EU201045 | X | French Guiana | Trinité | New | 04 35 00 N 53 21 00 W |
| *Adenomera andreae E* | 121AF | EU201049 | X | Suriname | Brownsberg | New | 04 56 31 N 55 10 33 W |
| *Adenomera heyeri A* | 221mc | EU201050 | X | French Guiana | Montagne des singes | New | 05 04 00 N 52 43 00 W |
| *Adenomera heyeri B* | 46PG | EU201051 | X | French Guiana | Piton baron | New | 03 17 00 N 53 04 00 W |
| *Adenomera hylaedactyla A* | 272mc | EU201054 | X | French Guiana | Kaw | New | 04 42 00 N 52 18 00 W |
| *Adenomera hylaedactyla B* | 92mc | EU201052 | X | French Guiana | Montagne d'argent | New | 04 23 00 N 51 42 00 W |
| *Adenomera hylaedactyla C* | 1235BPN | EU201053 | X | Guyana | Imbaimadai | New | 05 44 23 N 60 17 51 W |
| *Adenomera hylaedactyla D* | MJH 3669 | DQ283063 | X | Peru | Huanuco, Rio Llullapichis, Panguana | Frost et al., 2006 | 09 23 02 S 75 52 57 W |
| *Adenomera hylaedactyla E* | MZUSP 70958 | AY943240 | X | Brazil | Alter do Chão | de Sà et al., 2005 | 02 32 00 S 54 58 00 W |
| *Allobates femoralisA* | 56AF | EU201064 | X | French Guiana | Petit,saut | New | 05 04 00 N 53 03 00 W |
| *Allobates femoralisB* | 303MC | EU201065 | X | French Guiana | Toponowini | New | 03 03 10 N 52 42 37 W |
| *Allobates femoralisC* | LSUMZ 17552 | DQ283045 | X | Brazil | Rondonia, Rio Formoso, Parque Estadual Guajira,Mirim, approx. 90 km N Nova Mamore | Frost et al., 2006 | 10 19 17 S 64 33 47 W |
| *Allobates femoralisD* | OMNH 34568 | DQ502089 | O | Brazil | Para, 101 km S and 15 km E Santarem (near Rio Curua,Una) | Grant et al., 2006 | 03 09 00 S 54 50 00 W |
| *Allobates femoralisD* | OMNH 34572 | DQ502090 | O | Brazil | Para, 101 km S and 15 km E Santarem (near Rio Curua,Una) | Grant et al., 2006 | 03 09 00 S 54 50 00 W |
| *Allobates femoralisD* | MPEG 12021 | DQ502220 | X | Brazil | Para, 101 km S and 15 km E Santarem (near Rio Curua,Una) | Grant et al., 2006 | 03 09 00 S 54 50 00 W |
| *Allobates femoralisD* | MPEG 13415 | DQ502088 | O | Brazil | Rondonia, Parque Estadual Guajara,Mirim | Grant et al., 2006 | 10 19 17 S 64 33 47 W |
| *Allobates femoralisE* | MJH 3976 | DQ502113 | X | Brazil | Amazonas, Reserva Florestal Adolfo Ducke | Grant et al., 2006 | 02 58 51 S 59 55 16 W |
| *Allobates femoralisE* | AF124106 | AF124106 | O | Brazil | ? | Vences et al., 2003 | |
| *Allobates femoralisF* | MJH 7354 | DQ502117 | X | Peru | Huanuco, Rio Llullapichis, Panguana | Grant et al., 2006 | 09 23 02 S 75 52 57 W |
| *Allobates femoralisF* | AfemRSucv4a | DQ523023 | O | Peru | Rio Sucusari Iquitos, Loreto | Grant et al., 2006 | 03 14 26 N 72 55 42 W |
| *Allobates femoralisF* | AfemTahuiv1b | DQ523025 | O | Peru | Tahuayo River Iquitos, Loreto, | Grant et al., 2006 | 04 11 13 S 73 06 16 W |
| *Allobates femoralisF* | AfemShucv3a | DQ523072 | O | Peru | Shucshuyacu Yurimaguas, Loreto, | Roberts et al., 2006 | 06 00 59 S 75 50 40 W |
| *Allobates femoralisG* | OMNH 34102 | DQ502093 | O | Ecuador | Sucumbios, Estacion Cientifica de Universidad Catolica near Reserva Faunistica Cuyabeno, 220m | Grant et al., 2006 | 00 00 00 S 76 10 00 W |
| *Allobates femoralisG* | OMNH 34104 | DQ502094 | O | Ecuador | Sucumbios, Estacion Cientifica de Universidad Catolica near Reserva Faunistica Cuyabeno, 220m | Grant et al., 2006 | 00 00 00 S 76 10 00 W |
| *Allobates femoralisG* | LSU 12798 | DQ502228 | O | Ecuador | Sucumbios, Estacion Cientifica de Universidad Catolica near Reserva Faunistica Cuyabeno, 220m | Grant et al., 2006 | 00 00 00 S 76 10 00 W |
| *Allobates femoralisG* | QCAZ16484 | AY364543 | X | Ecuador | ? | Santos et al., 2003 | |
| *Allobates femoralisG* | | AF128572 | O | Ecuador | Cuyabeno | Vences et al., 2003 | 00 15 19 S 75 53 24 W |
| *Allobates femoralisH* | WED 55470; KU 205291 | AY326026 | X | Peru | Madre de Dios, Cusco Amazonico | Darst and Cannatella, 2003 | 11 39 00 S 70 33 35 W |
| *Allobates femoralisH* | WED 55560; KU 205292 | AY326027 | O | Peru | Madre de Dios, Cusco Amazonico | Darst and Cannatella, 2003 | 11 39 00 S 70 33 35 W |
| *Allobates femoralisH* | KU 215179 | DQ501990 | O | Peru | Madre de Dios, Cusco Amazonico, 15 km E Puerto Maldonado, 200 m | Grant et al., 2006 | 11 39 00 S 70 33 35 W |
| *Allobates femoralisH* | KU 215177 | DQ502014 | O | Peru | Madre de Dios, Cusco Amazonico, 15 km E Puerto Maldonado, 200 m | Grant et al., 2006 | 11 39 00 S 70 33 35 W |
| *Allobates femoralisH* | KU 215180 | DQ502015 | O | Peru | Madre de Dios, Cusco Amazonico, 15 km E Puerto Maldonado, 200 m | Grant et al., 2006 | 11 39 00 S 70 33 35 W |
| *Allobates femoralisH* | AfemBocMan22 | DQ523069 | O | Peru | Boca Manu Cuzco, | Roberts et al., 2006 | 12 16 32 S 70 56 49 W |
| *Allobates femoralisI* | OMNH 36066 | DQ502091 | O | Brazil | Acre, Porto Walter | Grant et al., 2006 | 08 15 31 S 72 46 37 W |
| *Allobates femoralisI* | OMNH 36070 | DQ502092 | X | Brazil | Acre, Porto Walter | Grant et al., 2006 | 08 15 31 S 72 46 37 W |
| *Allobates femoralisI* | OMNH 36073 | DQ502231 | O | Brazil | Acre, Porto Walter | Grant et al., 2006 | 08 15 31 S 72 46 37 W |
| *Allobates femoralisJ* | AfemRManv12 | DQ523040 | X | Peru | Rio Manati Iquitos, Loreto | Roberts et al., 2006 | |
| *Allobates femoralisK* | AfemItaya2ii | DQ523062 | O | Peru | Itaya River Iquitos, Loreto | Roberts et al., 2006 | 04 27 00 S 73 34 11 W |
| *Allobates femoralisK* | AfemNautv5a | DQ523059 | X | Peru | Nauta Road Iquitos, Loreto | Roberts et al., 2006 | 04 34 14 S 73 46 00 W |
| *Allobates femoralisL* | AfemMazukovi | DQ523055 | X | Peru | Mazuko Madre de Dios | Roberts et al., 2006 | |
| *Allobates femoralisM* | AfemSapoiv10 | DQ523082 | X | Peru | Saposoa Tarapoto, San Martin, | Roberts et al., 2006 | 06 46 15 S 76 56 28 W |

| | | | | | | | |
|---|---|---|---|---|---|---|---|
| *Allobates femoralisN* | UTA A56478 | DQ502246 | X | Suriname | Sipaliwini, in the vicinity of Kayser airstrip | Grant et al., 2006 | 03 05 70 N 56 28 30 W |
| *Allobates granti A* | 38RB | EU201067 | X | French Guiana | St Eugene | New | 04 51 00 N 53 04 00 W |
| *Allobates granti A* | MNHN???? | AY263233 | O | French Guiana | Saül | Vences et al., 2003 | 03 37 32 N 53 12 26 W |
| *Allobates granti B* | 185mc | EU201066 | X | French Guiana | Trijonction | New | 02 20 00 N 54 36 00 W |
| *Allobates granti C* | 148AF | EU201068 | X | Suriname | Brownsberg | New | 04 56 31 N 55 10 33 W |
| *Allobates granti D* | 49BM | EU201069 | X | French Guiana | Saül | New | 03 37 32 N 53 12 26 W |
| *Allobates granti D* | MNHN2000-651 | AY263230 | O | French Guiana | Saül | Vences et al., 2003 | 03 37 32 N 53 12 26 W |
| *Allobates granti D* | MNHN2000-653 | AY263232 | O | French Guiana | Saül | Vences et al., 2003 | 03 37 32 N 53 12 26 W |
| *Allobates sp.PEG-M3* | MPEG 13385 | DQ502191 | | Brazil | Rondonia, Parque Estadual Guajara,Mirim | Grant et al., 2006 | 10 19 17 S 64 33 47 W |
| *Allobates zaparo* | QCAZ16601 | AY364578 | | Ecuador | | Santos et al., 2003 | |
| *Allophryne ruthveni A* | 205mc | EU201098 | X | French Guiana | Cacao | New | 04 34 00 N 52 28 00 W |
| *Allophryne ruthveni B* | MAD 1512 | AY843564 | X | Guyana | Kabocali camp, 101 m | Faivovich et al., 2005 | 04 17 10 N 58 30 56 W |
| *Allophryne ruthveni C* | ? | AF364512 | X | Brazil | eastern Amazon near Rio Xingu, | Austin et al., 2001 | 07 39 00 S 51 21 00 W |
| *Ameerega hahneli A* | MNHN 2000-656 | AY263247 | X | French Guiana | Trinité | Vences et al., 2003 | 04 35 00 N 53 21 00 W |
| *Ameerega hahneli B* | OMNH 36090 | DQ502085 | O | Brazil | Acre, Porto Walter | Grant et al., 2006 | 08 15 31 S 72 46 37 W |
| *Ameerega hahneli B* | OMNH 37443 | DQ502081 | O | Brazil | Amazonas, Castanho, ca. 40 km S Manaus,at km 12 on road to Autazes | Grant et al., 2006 | 03 37 10 S 59 86 78 W |
| *Ameerega hahneli B* | MPEG 13849 | DQ502086 | O | Brazil | Amazonas, Castanho, ca. 40 km S Manaus,at km 12 on road to Autazes | Grant et al., 2006 | 03 37 10 S 59 86 78 W |
| *Ameerega hahneli B* | OMNH 37444 | DQ502087 | X | Brazil | Amazonas, Castanho, ca. 40 km S Manaus,at km 12 on road to Autazes | Grant et al., 2006 | 03 37 10 S 59 86 78 W |
| *Ameerega hahneli B* | MPEG 13844 | DQ502226 | O | Brazil | Amazonas, Castanho, ca. 40 km S Manaus,at km 12 on road to Autazes | Grant et al., 2006 | 03 37 10 S 59 86 78 W |
| *Ameerega hahneli B* | EhahnAmazvii1b | DQ523063 | O | Brazil | Amazonas | Roberts et al., 2006 | |
| *Ameerega hahneli B* | EhahnAmaz1E | DQ523067 | O | Brazil | Amazonas | Roberts et al., 2006 | |
| *Ameerega hahneli C* | OMNH 36088 | DQ502077 | X | Brazil | Acre, Porto Walter | Grant et al., 2006 | 08 15 31 S 72 46 37 W |
| *Ameerega hahneli C* | MPEG 12420 | DQ502083 | O | Brazil | Acre, Porto Walter | Grant et al., 2006 | 08 15 31 S 72 46 37 W |
| *Ameerega hahneli D* | OMNH 36092 | DQ502084 | X | Brazil | Acre, Porto Walter | Grant et al., 2006 | 08 15 31 S 72 46 37 W |
| *Ameerega hahneli D* | EhahnPWvii1a | DQ523064 | O | Brazil | Acre Porto Walter | Roberts et al., 2006 | 08 15 31 S 72 46 37 W |
| *Ameerega hahneli E* | | AF282246 | X | Bolivia | Cobija | Loetters and Vences, 2000 | 11 01 12 S 68 46 00 W |
| *Ameerega hahneli E* | KU 215183 | DQ501991 | O | Peru | Madre de Dios, Cusco Amazonico, 15 km E Puerto Maldonado, 200 m | Grant et al., 2006 | 11 39 00 S 70 33 35 W |
| *Ameerega hahneli E* | KU 215185 | DQ501996 | X | Peru | Madre de Dios, Cusco Amazonico, 15 km E Puerto Maldonado, 200 m | Grant et al., 2006 | 11 39 00 S 70 33 35 W |
| *Ameerega hahneli E* | KU 215184 | DQ501997 | O | Peru | Madre de Dios, Cusco Amazonico, 15 km E Puerto Maldonado, 200 m | Grant et al., 2006 | 11 39 00 S 70 33 35 W |
| *Ameerega hahneli E* | EhahnAP8iii9b | DQ523034 | O | Peru | Alto Purus River Ucayali | Roberts et al., 2006 | 09 24 05 S 73 15 29 W |
| *Ameerega hahneli E* | EhahnAPR8iii1c | DQ523041 | O | Peru | Alto Purus River Ucayali | Roberts et al., 2006 | 09 24 05 S 73 15 29 W |
| *Ameerega hahneli E* | EhahnAP8iii9a | DQ523081 | O | Peru | Alto Purus River Ucayali | Roberts et al., 2006 | 09 24 05 S 73 15 29 W |
| *Ameerega hahneli E* | EhahnBocMan | DQ523027 | O | Peru | Boca Manu Cuzco | Roberts et al., 2006 | 12 16 32 S 70 56 49 W |
| *Ameerega hahneli E* | EhahnRAmigosix | DQ523056 | O | Peru | Rio Amigos Madre de Dios | Roberts et al., 2006 | |
| *Ameerega hahneli F* | | AF282248 | X | Peru | Huanuco, Rio Llullapichis,Panguana | Loetters and Vences, 2000 | 09 23 02 S 75 52 57 W |
| *Ameerega hahneli G* | QCAZ13325 | AY364573 | X | Ecuador | | Santos et al., 2003 | |
| *Ameerega hahneli H* | ICN 50410 | DQ502270 | X | Colombia | Amazonas, Leticia, Lago Yahuarcaca | Grant et al., 2006 | 04 10 06 S 69 54 41 W |
| *Ameerega hahneli H* | EhahnConvviii4 | DQ523032 | O | Peru | Convento Tarapoto, San Martin | Roberts et al., 2006 | 06 15 03 S 76 18 52 W |
| *Ameerega hahneli H* | EhahnItayaiii2 | DQ523033 | X | Peru | Itaya River Iquitos, Loreto | Roberts et al., 2006 | 04 27 00 S 73 34 11 W |
| *Ameerega hahneli H* | EhahnItaya2iii | DQ523061 | O | Peru | Itaya River Iquitos, Loreto | Roberts et al., 2006 | 04 27 00 S 73 34 11 W |
| *Ameerega hahneli I* | EhahnIvochviii | DQ523038 | X | Peru | Ivochote Cuzco | Roberts et al., 2006 | 12 28 15 S 72 59 37 W |
| *Ameerega hahneli J* | EhahnAguamiii3 | DQ523037 | X | Peru | Aguamo,Muyuma Tarapoto, San Martin | Roberts et al., 2006 | 06 30 42 S 76 28 54 W |
| *Ameerega hahneli K* | EhahnSapoiii9d | DQ523086 | X | Peru | Saposoa Tarapoto, San Martin | Roberts et al., 2006 | 06 46 15 S 76 56 28 W |
| *Ameerega hahneli L* | | DQ523022 | O | Peru | Cachiyacu Road Tarapoto, San Martin | Roberts et al., 2006 | 06 30 42 S 76 28 54 W |
| *Ameerega hahneli L* | EhahnTCRdi6a | DQ523026 | X | Peru | Cachiyacu Road Tarapoto, San Martin | Roberts et al., 2006 | 06 28 39 S 76 19 21 W |
| *Ameerega hahneli L* | EhahnTCRd1 | DQ523078 | O | Peru | Cachiyacu Road Tarapoto, San Martin | Roberts et al., 2006 | 06 28 39 S 76 19 21 W |
| *Ameerega hahneli L* | EhahnChaz2B | DQ523051 | O | Peru | Near Chazuta Tarapoto, San Martin | Roberts et al., 2006 | 06 58 00 S 76 15 00 W |

| | | | | | | | |
|---|---|---|---|---|---|---|---|
| *Ameerega hahneli L* | TSRdviii2 | DQ523079 | O | Peru | Road to Sisa Tarapoto, San Martin | Roberts et al., 2006 | |
| *Ameerega hahneli L* | EhahnVSAiv3a | DQ523049 | O | Peru | Valle San Antonio Tarapoto, San Martin | Roberts et al., 2006 | |
| *Ameerega hahneli M* | EhahnRManv9a | DQ523075 | X | Peru | Loreto, Iquitos Rio Manati | Roberts et al., 2006 | |
| *Ameerega trivittata A* | MPEG 12504 | DQ502079 | X | Brazil | Acre, Porto Walter | Grant et al., 2006 | 08 15 31 S 72 46 37 W |
| *Ameerega trivittata A* | EtrivAmazvii1d | DQ523065 | O | Brazil | Amazonas | Grant et al., 2006 | |
| *Ameerega trivittata A* | MJH 3907 | DQ502112 | O | Brazil | Amazonas, Base 2 island in reservoir of Uatuma river, 8 km NW represa de Balbina | Grant et al., 2006 | 01 50 40 S 59 33 43 W |
| *Ameerega trivittata A* | OMNH 37453 | DQ502148 | X | Brazil | Amazonas, Castanho, ca. 40 km S Manaus, at km 12 on road to Autazes | Grant et al., 2006 | 03 37 10 S 59 86 78 W |
| *Ameerega trivittata A* | OMNH 37455 | DQ502147 | O | Brazil | Amazonas, Castanho, ca. 40 km S Manaus, at km 12 on road to Autazes | Roberts et al., 2006 | 03 37 10 S 59 86 78 W |
| *Ameerega trivittata A* | ??????????? | DQ523077 | X | French Guiana | ???????????????????? | Roberts et al., 2006 | |
| *Ameerega trivittata A* | BPN 910 | DQ502250 | X | Suriname | Road to Apura | Grant et al., 2006 | 05 11 00 N 55 37 00 W |
| *Ameerega trivittata B* | USNM 268846 | DQ502021 | X | Peru | Madre de Dios, Puerto Maldonado, Explorer's Inn, 30 km (airline) SSW of Tambopata Reserve | Grant et al., 2006 | 12 35 60 S 69 10 60 W |
| *Ameerega trivittata B* | USNM 269052 | DQ502023 | O | Peru | Madre de Dios, Puerto Maldonado, Explorer's Inn, 30 km (airline) SSW of Tambopata Reserve | Grant et al., 2006 | 12 35 60 S 69 10 60 W |
| *Ameerega trivittata C* | | AF098750 | O | ? | ? | Summers et al., 1999 | |
| *Ameerega trivittata C* | ZFMK 69880 | AF124128 | O | captive | ? | Vences et al., 2003 | |
| *Ameerega trivittata C* | | DQ523068 | X | Peru | Cordillera Oriental Amazonas, | Roberts et al., 2006 | 10 26 00 S 74 31 00 W |
| *Ameerega trivittata C* | | DQ523021 | O | Peru | Shilcayo Valley Tarapoto, San Martin | Roberts et al., 2006 | |
| *Ameerega trivittata C* | | AF128569 | O | Peru, | Yurimaguas | Vences et al., 2003 | 05 55 05 S 76 05 40 W |
| *Ameerega trivittata D* | MPEG 12450 | DQ502082 | O | Brazil | Acre, Porto Walter | Grant et al., 2006 | 08 15 31 S 72 46 37 W |
| *Ameerega trivittata D* | MPEG 12447 | DQ502219 | O | Brazil | Acre, Porto Walter | Grant et al., 2006 | 08 15 31 S 72 46 37 W |
| *Ameerega trivittata D* | MPEG 12468 | DQ502227 | X | Brazil | Acre, Porto Walter | Grant et al., 2006 | 08 15 31 S 72 46 37 W |
| *Ameerega trivittata D* | | DQ523029 | O | Brazil | Acre, Porto Walter | Roberts et al., 2006 | 08 15 31 S 72 46 37 W |
| *Ameerega trivittata D* | ICN 50437 | DQ502267 | X | Colombia | Amazonas, Leticia, Km 11 (Leticia,Tarapaca) | Grant et al., 2006 | 04 10 06 S 69 54 41 W |
| *Ameerega trivittata D* | | DQ523028 | O | Peru | Alto Purus River Ucayali, | Roberts et al., 2006 | 09 24 05 S 73 15 29 W |
| *Ameerega trivittata D* | | DQ523030 | O | Peru | Chumilla San Martin, | Roberts et al., 2006 | 05 51 23 S 77 02 10 W |
| *Ameerega trivittata D* | | DQ523050 | X | Peru | Cordillera Azul San Martin, | Roberts et al., 2006 | 07 26 24 S 76 57 03 W |
| *Ameerega trivittata D* | | DQ523046 | O | Peru | Iscozazin Pasco | Roberts et al., 2006 | 10 11 19 S 75 09 37 W |
| *Ameerega trivittata D* | | DQ523054 | O | Peru | Near Bonilla Tarapoto, San Martin | Roberts et al., 2006 | 07 02 46 S 76 47 00 W |
| *Ameerega trivittata D* | | DQ523071 | O | Peru | Near Chazuta Tarapoto, San Martin | Roberts et al., 2006 | 06 58 00 S 76 15 00 W |
| *Ameerega trivittata D* | | DQ523031 | O | Peru | Rio Manati Iquitos, Loreto | Roberts et al., 2006 | |
| *Ameerega trivittata D* | | DQ523058 | O | Peru | Road to Barranquita Tarapoto, San Martin, | Roberts et al., 2006 | |
| *Ameerega trivittata D* | | DQ523047 | O | Peru | Santa Rosa Huanuco | Roberts et al., 2006 | 08 50 12 S 74 34 17 W |
| *Ameerega trivittata D* | | DQ523035 | O | Peru | Tahuayo River Iquitos, Loreto, | Roberts et al., 2006 | 04 11 13 S 73 06 16 W |
| *Ameerega trivittata D* | | DQ523052 | O | Peru | Tahuayo River Iquitos, Loreto, | Roberts et al., 2006 | 04 11 13 S 73 06 16 W |
| *Ameerega trivittata D* | | DQ523066 | O | Peru | Tahuayo River Iquitos, Loreto, | Roberts et al., 2006 | 04 11 13 S 73 06 16 W |
| *Ameerega trivittata D* | LM 739-A | U39973 | O | Peru, | Huanuco, Rio Llullapichis,Panguana | Ruvinski and maxson, 1996 | 09 23 02 S 75 52 57 W |
| *Ameerega trivittata E* | | DQ523036 | X | Peru | Rio Sucusari Iquitos, Loreto | Roberts et al., 2006 | 03 14 26 N 72 55 42 W |
| *Ameerega trivittata F* | MJH 7483 | DQ502111 | X | Peru | Huanuco, Rio Llullapichis, Panguana | Grant et al., 2006 | 09 23 02 S 75 52 57 W |
| *Amerega macero* | LR 742 | DQ502155 | | Peru | Madre de Dios, Parque Nacional del Manu | Grant et al., 2006 | 12 15 00 S 71 45 00 W |
| *Anomaloglossus baeobatrachus A* | 220MC | EU201070 | X | French Guiana | Saül | New | 03 37 32 N 53 12 26 W |
| *Anomaloglossus baeobatrachus A* | MNHN1995-9454 | AY263236 | O | French Guiana | Aratai | Vences et al., 2003 | 05 10 00 N 54 20 00 W |
| *Anomaloglossus baeobatrachus A* | MNHN2000-0654 | AY263231 | O | French Guiana | Saül | Vences et al., 2003 | 03 37 32 N 53 12 26 W |
| *Anomaloglossus baeobatrachus B* | 148mc | EU201072 | X | French Guiana | Trijonction | New | 02 20 00 N 54 36 00 W |
| *Anomaloglossus baeobatrachus C* | 182mc | EU201071 | X | French Guiana | Trijonction | New | 02 20 00 N 54 36 00 W |
| *Anomaloglossus baeobatrachus D* | 149AF | | X | Suriname | Brownsberg | New | 04 56 31 N 55 10 33 W |
| *Anomaloglossus degranvillei A* | 17RB | EU201076 | X | French Guiana | St Eugene | New | 04 51 00 N 53 04 00 W |
| *Anomaloglossus degranvillei B* | 355MC | EU201075 | X | French Guiana | Lucifer | New | 04 46 00 N 53 55 00 W |
| *Anomaloglossus degranvillei C* | 68MC | EU201073 | X | French Guiana | Monts Bakra | New | 03 18 08 N 52 56 73 W |

| | | | | | | | |
|---|---|---|---|---|---|---|---|
| *Anomaloglossus degranvillei D* | 125BM | EU201074 | X | French Guiana | Saül | New | 03 37 32 N 53 12 26 W |
| *Anomaloglossus degranvillei D* | MNHN2000-0655 | AY263234 | O | French Guiana | Saül | Vences et al., 2003 | 03 37 32 N 53 12 26 W |
| *Anomaloglossus degranvillei E* | 143AF | EU201078 | X | Suriname | Brownsberg | New | 04 56 31 N 55 10 33 W |
| *Anomaloglossus degranvillei F* | 3025T | EU201077 | X | French Guiana | Mitaraka | New | 02 16 00 N 54 31 00 W |
| *Anomaloglossus degranvillei G* | 113MC | EU201079 | X | French Guiana | Tibourou | New | 04 25 00 N 52 18 00 W |
| *Anomaloglossus degranvillei H* | 230MC | EU201080 | X | French Guiana | Saül | New | 03 37 32 N 53 12 26 W |
| *Anomaloglossus degranvillei I* | 38AF | EU201081 | X | French Guiana | Saül | New | 03 37 32 N 53 12 26 W |
| *Anomaloglossus degranvillei J* | 278 | DQ502019 | X | Guyana | Mereme Mountains | Grant et al., 2006 | |
| *Anomaloglossus degranvillei K* | CPI 10209 | DQ502257 | X | Guyana | Mazaruni,Potoro, Mt. Roraima, 1075 m | Grant et al., 2006 | 01 49 19 N 61 52 15 W |
| *Anomaloglossus sp. "ayanganna"* | ROM39639 | DQ502129 | | Guyana | Mt Ayanganna | Grant et al., 2006 | 5 23 00 N 59 59 00 W |
| *Anomaloglossus sp. Tafelberg* | UTA A55758 | DQ502247 | | Suriname | Sipaliwini, ca. 4.0 km N of Tafelberg airstrip | Grant et al., 2006 | 03 47 00 N 56 09 00 W |
| *Anomaloglossus sp.Brownsberg D* | UTA A56469 | DQ502249 | | Suriname | Brokopondo, Brownsberg Nature Park | Grant et al., 2006 | 04 43 00 N 56 13 00 W |
| *Chaunus granulosus A* | USNM302451 | AY680261 | O | Brazil | Roraima, Caracarann | Pauly et al., 2004 | 03 50 00 N 59 47 00 W |
| *Chaunus granulosus A* | USNM302450 | DQ158457 | X | Brazil | Roraima | Pramuk, 2007 | 03 50 00 N 59 47 00 W |
| *Chaunus granulosus A* | 235mc | EF364306 | X | French Guiana | Mana | Fouquet et al., 2007 | 05 39 00 N 53 47 00 W |
| *Chaunus granulosus A* | AMNH A139020 | DQ283332 | X | Guyana | Southern Rupununi Savanna, Aishalton (onKubabawau Creek), 150 m | Frost et al., 2006 | 02 28 31 N 59 19 16 W |
| *Chaunus granulosus A* | 185AF | EU201056 | X | Suriname | Brownsberg | New | 04 43 00 N 56 13 00 W |
| *Chaunus granulosus B* | LM 1493 | AY028483 | X | Brazil | Rondonia, Porto Velho | Pramuk, 2001 | 08 44 38 S 64 04 59 N |
| *Chaunus granulosus B* | LM 1493 | AY028496 | O | Brazil | Rondonia, Porto Velho | Pramuk, 2001 | 08 44 38 S 64 04 59 N |
| *Chaunus granulosus C* | AF0093 | DQ158458 | X | Brazil | Roraima | Pramuk, 2006 | 03 50 00 N 59 47 00 W |
| *Chiasmocleis hudsoni A* | 28bm | EU201099 | X | French Guiana | Monts Bakra | New | 03 18 08 N 52 56 73 W |
| *Chiasmocleis hudsoni B* | 439PG | EU201100 | X | French Guiana | Kotika | New | 03 56 05 N 54 12 17 W |
| *Ctenophryne geayi* | 21bm | EU201103 | X | French Guiana | Trinité | New | 04 35 00 N 53 21 00 W |
| *Ctenophryne geayi* | AMNH A166444 | DQ283383 | X | Guyana | Berbice River camp at ca. 18 mi (linear) SW Kwakwani (ca. 2 mi downriver from Kurundi River confluence), 200 ft | Frost et al., 2006 | 05 05 06 N 58 14 14 W |
| *Dendrobates azureus* | CFBH 4203 | AY263250 | | Brazil | uncertain possibly Alto rio trombetas | Vences et al., 2003 | |
| *Dendrobates tinctorius A* | 1mc | EU201082 | X | French Guiana | Ouanary | New | 04 15 00 N 51 40 00 W |
| *Dendrobates tinctorius B* | UTA A56495 | DQ502248 | X | Suriname | Sipaliwini, ca. 1.0 km N of Tafelberg airstrip | Grant et al., 2006 | 03 47 00 N 56 09 00 W |
| *Dendrophryniscus minutus A* | 98bm | EF364310 | X | French Guiana | Mt Arawa | Fouquet et al., 2007 | 02 48 59 N 53 21 59 W |
| *Dendrophryniscus minutus B* | 3035T | EU201057 | X | French Guiana | Mitaraka | New | 02 16 00 N 54 31 00 W |
| *Dendrophryniscus minutus C* | QCAZ13965 | AF375516 | X | Ecuador | ? | Gluesenkamp, unpublished | |
| *Dendrophryniscus minutus D* | QCAZ 883 | DQ158420 | X | Ecuador | ? | Pramuk, 2006 | |
| *Dendrophryniscus minutus E* | MJH7095 | AY843582 | X | Peru | Huanuco, Rio Llullapichis, Panguana | Faivovich et al., 2005 | 09 23 02 S 75 52 57 W |
| *Dendropsophus brevifrons* | 28mc | EF376058 | X | French Guiana | Kaw | Salducci et al., 2005 | 04 42 00 N 52 18 00 W |
| *Dendropsophus brevifrons* | MJH 7101 | AY843611 | X | Peru | Huanuco, Rio Llullapichis, Panguana | Faivovich et al., 2005 | 09 23 02 S 75 52 57 W |
| *Dendropsophus leucophyllatus A* | Man-95231 | AF308091 | O | Brazil | Manaus, Amazonas | Chek et al., 2001 | 02 28 00 S 60 00 57 W |
| *Dendropsophus leucophyllatus A* | Man-95232 | AF308092 | X | Brazil | Manaus, Amazonas | Chek et al., 2001 | 02 28 00 S 60 00 57 W |
| *Dendropsophus leucophyllatus A* | SdN-95143 | AF308087 | O | Brazil | Serra do Navio, Amapa | Chek et al., 2001 | 00 55 05 N 52 00 10 W |
| *Dendropsophus leucophyllatus A* | SdN-95156 | AF308088 | X | Brazil | Serra do Navio, Amapa | Chek et al., 2001 | 00 55 05 N 52 00 10 W |
| *Dendropsophus leucophyllatus A* | 95161 | DQ393416 | O | Brazil | Alter do Chão | Lougheed et al., 2006 | 02 32 00 S 54 58 00 W |
| *Dendropsophus leucophyllatus A* | 95232 | DQ393427 | O | Brazil | approx. 100 km north of Manaus, Amazonas | Lougheed et al., 2006 | 02 28 00 S 60 00 57 W |
| *Dendropsophus leucophyllatus A* | 95193 | DQ393428 | O | Brazil | approx. 100 km north of Manaus, Amazonas | Lougheed et al., 2006 | 02 28 00 S 60 00 57 W |
| *Dendropsophus leucophyllatus A* | 95143 | DQ393417 | O | Brazil | Serra do Navio, Amapa | Lougheed et al., 2006 | 00 55 05 N 52 00 10 W |
| *Dendropsophus leucophyllatus A* | 36mc | EF376059 | X | French Guiana | Kaw | Salducci et al., 2005 | 04 42 00 N 52 18 00 W |
| *Dendropsophus leucophyllatus B* | R.Bran-95253 | AF308097 | O | Brazil | Rio Branco, Acre | Chek et al., 2001 | 09 58 00 S 67 48 00 W |
| *Dendropsophus leucophyllatus B* | 93044 | DQ393421 | X | Brazil | approx. 200km west of Redenção, Para | Lougheed et al., 2006 | 07 40 00 S 51 22 00 W |
| *Dendropsophus leucophyllatus B* | 95253 | DQ393432 | X | Brazil | Rio Branco, Acre | Lougheed et al., 2006 | 09 58 00 S 67 48 00 W |
| *Dendropsophus leucophyllatus B* | 95254 | DQ393433 | O | Brazil | Rio Branco, Acre | Lougheed et al., 2006 | 09 58 00 S 67 48 00 W |

| | | | | | | | |
|---|---|---|---|---|---|---|---|
| *Dendropsophus leucophyllatus C* | AdC-95163 | AF308089 | X | Brazil | Alter do Chão | Chek et al., 2001 | 02 33 00 S 54 59 00 W |
| *Dendropsophus leucophyllatus C* | 95163 | DQ393419 | O | Brazil | Alter do Chão | Lougheed et al., 2006 | 02 32 00 S 54 58 00 W |
| *Dendropsophus leucophyllatus D* | 95162 | DQ393422 | X | Brazil | Alter do Chão | Lougheed et al., 2006 | 02 32 00 S 54 58 00 W |
| *Dendropsophus leucophyllatus E* | Aukre-93045 | AF308085 | X | Brazil | A,Ukre, Para | Chek et al., 2001 | 07 40 00 S 51 22 00 W |
| *Dendropsophus leucophyllatus E* | Aukre-93046 | AF308086 | O | Brazil | A,Ukre, Para | Chek et al., 2001 | 07 40 00 S 51 22 00 W |
| *Dendropsophus leucophyllatus E* | 93042, 93043 | DQ393418 | O | Brazil | approx. 200km west of Redenção, Para | Lougheed et al., 2006 | 07 40 00 S 51 22 00 W |
| *Dendropsophus leucophyllatus F* | 93049 | DQ393420 | X | Brazil | approx. 200km west of Redenção, Para | Lougheed et al., 2006 | 07 40 00 S 51 22 00 W |
| *Dendropsophus leucophyllatus G* | Jur-4483 INPA4483 | AF308096 | O | Brazil | Nova Vida, Acre | Chek et al., 2001 | 08 35 00 S 72 50 00 W |
| *Dendropsophus leucophyllatus G* | Obd-95176 | AF308090 | X | Brazil | Obidos, Pará | Chek et al., 2001 | 01 55 00 S 55 31 00 W |
| *Dendropsophus leucophyllatus G* | Jur-4273 INPA4273 | AF308095 | X | Brazil | Porongaba, Acre | Chek et al., 2001 | 08 41 00 S 72 48 00 W |
| *Dendropsophus leucophyllatus G* | Tab-96056 | AF308094 | O | Brazil | Tabatinga, Amazonas | Chek et al., 2001 | 04 16 00 S 69 58 00 W |
| *Dendropsophus leucophyllatus G* | 4273 | DQ393431 | O | Brazil | Igarapé Porongaba, Acre | Lougheed et al., 2006 | 08 40 00 S 72 47 00 W |
| *Dendropsophus leucophyllatus G* | 4483 | DQ393430 | O | Brazil | Nova Vida, Acre | Lougheed et al., 2006 | 08 22 00 S 72 49 00 W |
| *Dendropsophus leucophyllatus G* | 95175 | DQ393423 | O | Brazil | Obidos, Pará | Lougheed et al., 2006 | 01 55 00 S 55 31 00 W |
| *Dendropsophus leucophyllatus G* | 95176 | DQ393424 | O | Brazil | Obidos, Pará | Lougheed et al., 2006 | 01 55 00 S 55 31 00 W |
| *Dendropsophus leucophyllatus G* | 95178 | DQ393425 | O | Brazil | Obidos, Pará | Lougheed et al., 2006 | 01 55 00 S 55 31 00 W |
| *Dendropsophus leucophyllatus G* | 95172 | DQ393426 | O | Brazil | Obidos, Pará | Lougheed et al., 2006 | 01 55 00 S 55 31 00 W |
| *Dendropsophus leucophyllatus G* | Tab-96018 | AF308093 | O | Brazil | Tabatinga, Amazonas | Lougheed et al., 2006 | 04 16 00 S 69 58 00 W |
| *Dendropsophus leucophyllatus G* | 96023 | DQ393429 | O | Brazil | Tabatinga, Amazonas | Lougheed et al., 2006 | 04 16 00 S 69 58 00 W |
| *Dendropsophus leucophyllatus G* | 96034 | DQ393434 | O | Brazil | Tabatinga, Amazonas | Lougheed et al., 2006 | 04 16 00 S 69 58 00 W |
| *Dendropsophus leucophyllatus G* | 96009, 96018 | DQ393435 | O | Brazil | Tabatinga, Amazonas | Lougheed et al., 2006 | 04 16 00 S 69 58 00 W |
| *Dendropsophus leucophyllatus G* | 96019, 96020 | DQ393436 | O | Brazil | Tabatinga, Amazonas | Lougheed et al., 2006 | 04 16 00 S 69 58 00 W |
| *Dendropsophus minutus A* | 114mc | EF376063 | X | French Guiana | Kaw | Salducci et al., 2005 | 04 42 00 N 52 18 00 W |
| *Dendropsophus minutus B* | MZUSP70297 | AF308112 | O | Brazil | A,Ukre, Para | Chek et al 01 | 07 40 00 S 51 22 00 W |
| *Dendropsophus minutus B* | MZUSP70296 | AF308113 | X | Brazil | A,Ukre, Para | Chek et al 01 | 07 40 00 S 51 22 00 W |
| *Dendropsophus minutus C* | MACN 33799 | AY549345 | X | Argentina | Misiones, Guarani, San Vicente, Campo Anexo INTA 'Cuartel Rio Victoria | Faivovich et al., 2004 | 26 56 00 S 54 24 00 W |
| *Dendropsophus nanus A* | 170bm | EU201104 | X | French Guiana | Kaw | New | 04 42 00 N 52 18 00 W |
| *Dendropsophus nanus A* | 84mc | EF376063 | O | French Guiana | Kaw | Salducci et al., 2005 | 04 42 00 N 52 18 00 W |
| *Dendropsophus nanus B* | MACN 37785 | AY549346 | X | Argentina | Entre Rios, Dto. Islas del Ibicuy | Faivovich et al., 2004 | 32 07 02 S 59 18 15 W |
| *Dendropsophus triangulum* | KU202745 | AY326053 | | Ecuador | Napo, Misahualli, 600 m | Darst and Cannatella, 2003 | 01 02 00 S 77 40 13 W |
| *Dendropsophus walfordi* | MJH 129 | AY843683 | | Brazil | ? | Faivovich et al., 2005 | |
| *Elachistocleis ovalis* | 82mc | EU201101 | X | French Guiana | Montjoly | New | 04 55 00 N 52 16 00 W |
| *Elachistocleis ovalis* | AMNH A141136 | DQ283405 | X | Guyana | Dubulay Ranch on the Berbice River, 200ft | Frost et al 06 | 05 40 55 N 57 51 32 W |
| *Eleutherodactylus chiastonotus A* | 101mc | EU201060 | X | French Guiana | Tibourou | New | 04 25 00 N 52 18 00 W |
| *Eleutherodactylus chiastonotus B* | 162AF | EU201061 | X | Suriname | Brownsberg | New | 04 56 31 N 55 10 33 W |
| *Eleutherodactylus marmoratus A* | 110bm | EU201063 | X | French Guiana | Kaw | New | 04 42 00 N 52 18 00 W |
| *Eleutherodactylus marmoratus B* | 77mc | EU201062 | X | French Guiana | Trois saut | New | 02 14 00 N 52 52 00 W |
| *Eleutherodactylus zeuctotylus A* | 95mc | EF376083 | X | French Guiana | Tibourou | Salducci et al., 2005 | 04 25 00 N 52 18 00 W |
| *Eleutherodactylus zeuctotylus B* | 100AF | EU201059 | X | Suriname | Road to Apura | New | 05 11 00 N 55 39 00 W |
| *Engystomops petersi* | QCAZ 11965 | DQ337231 | O | Ecuador | Napo, Cando | Ron et al., 2006 | |
| *Engystomops petersi* | QCAZ 14723 | DQ337232 | O | Ecuador | Napo, Napo,Galeras, Ishquiñambi | Ron et al., 2006 | |
| *Engystomops petersi* | QCAZ 12128 | DQ337233 | O | Ecuador | Orellana, Estación Científica Yasuní, Universidad Católica del Ecuador | Ron et al., 2006 | 00 40 00 S 76 23 00 W |
| *Engystomops petersi A* | 108bm | EU201097 | X | French Guiana | Trinité | New | 04 35 00 N 53 21 00 W |
| *Engystomops petersi B* | QCAZ 23976 | DQ337234 | X | Ecuador | Sucumbíos, La Selva | Ron et al., 2006 | 00 00 33 S 76 35 23 W |
| *Engystomops petersi C* | QCAZ 26210 | DQ337230 | X | Ecuador | Pastaza, El Puyo | Ron et al., 2006 | 01 29 04 S 78 00 38 W |
| *Hamptophryne boliviana A* | | AF215370 | O | ????? | ??????????????????????????????????? | Vences, 2000 | |
| *Hamptophryne boliviana A* | 104bm | EU201102 | X | French Guiana | Trinité | New | 04 35 00 N 53 21 00 W |

| | | | | | | | |
|---|---|---|---|---|---|---|---|
| *Hamptophryne boliviana B* | Rafael de Sa | DQ283438 | X | Peru | ? | Frost et al., 2006 | |
| *Hypsiboas boans A* | 43mc | EU201105 | X | French Guiana | Kaw | New | 04 42 00 N 52 18 00 W |
| *Hypsiboas boans A* | 99bm | EF376055 | | French Guiana | Monts Bakra | Salducci et al., 2005 | 03 18 08 N 52 56 73 W |
| *Hypsiboas boans A* | RWM 17746 | AY843610 | X | Venezuela | Amazonas, Cano Agua Blanca, 3.5 Km SE Neblina Base Camp on Rio Mawarinuma (=Rio Baria) | Faivovich et al., 2005 | 00 49 50 N 66 09 40 W |
| *Hypsiboas calcaratus A* | 131mc | EF376064 | X | French Guiana | Crique margot | Salducci et al., 2005 | 05 28 00 N 53 57 00 W |
| *Hypsiboas calcaratus B* | NMP6V 71250 | AY843613 | X | Peru | Anguilla, 50 km W of Iquitos | Faivovich et al., 2005 | 03 43 47 S 73 49 51 W |
| *Hypsiboas calcaratus C* | WED 54086; KU 202911 | AY326056 | X | Ecuador | Napo, Misahualli, 600 m | Darst and Cannatella, 2003 | 01 02 00 S 77 40 13 W |
| *Hypsiboas crepitans A* | 95bm | EU201107 | X | French Guiana | Mont St Marcel | New | 02 23 09 N 53 00 68 W |
| *Hypsiboas crepitans B* | CFBH2966 | AY843621 | X | Brazil | Alagoas, Municipio de Piranhas, Represa de Xingo | Faivovich et al., 2005 | 09 39 22 S 36 42 08 W |
| *Hypsiboas fasciatus A* | 229mc | EU201108 | X | French Guiana | Saül | New | 03 37 32 N 53 12 26 W |
| *Hypsiboas fasciatus A* | AMNH-A 164081 | AY549335 | X | Guyana | Iwokrama, Cowfly camp | Faivovich et al., 2004 | 04 40 17 N 58 41 06 W |
| *Hypsiboas fasciatus A* | 97AF | EU201109 | X | Suriname | Road to Apura | New | 05 11 00 N 55 39 00 W |
| *Hypsiboas fasciatus B* | 168mc | EF376065 | X | French Guiana | Guatemala | Salducci et al., 2005 | 05 09 00 N 52 38 00 W |
| *Hypsiboas geographicus A* | 33mc | EF376054 | X | French Guiana | Grand,Santi | Salducci et al., 2005 | 04 20 00 N 54 15 00 W |
| *Hypsiboas geographicus A* | AMNH-A 141054; AMCC 101481 | AY843628 | X | Guyana | Warniabo Creek, 4 mi (by rd) SW Dubulay Ranch house | Faivovich et al., 2005 | 05 37 56 N 57 53 55 W |
| *Hypsiboas geographicus B* | 171bm | EU201106 | X | French Guiana | Trinité | New | 04 35 00 N 53 21 00 W |
| *Hypsiboas granosus A* | 189bm | EU201113 | X | French Guiana | Trinité | New | 04 35 00 N 53 21 00 W |
| *Hypsiboas granosus B* | AMNH-A 164105 | AY549336 | X | Guyana | Iwokrama, Muri Scrub camp | Faivovich et al., 2004 | 04 40 17 N 58 41 06 W |
| *Hypsiboas multifasciatus A* | 241mc | EU201111 | X | French Guiana | Saül | New | 03 37 32 N 53 12 26 W |
| *Hypsiboas multifasciatus A* | 38mc | EF376057 | | French Guiana | Kaw | Salducci et al., 2005 | 04 42 00 N 52 18 00 W |
| *Hypsiboas multifasciatus B* | 47AF | EU201110 | X | French Guiana | Petit,saut | New | 05 04 00 N 53 03 00 W |
| *Hypsiboas multifasciatus B* | AMNH A-141040; AMCC 101446 | AY843648 | X | Guyana | Demerara, Ceiba Station, Madewini River, ca 3 mi (linear) E Timehri Airport | Faivovich et al., 2005 | 06 28 24 N 58 03 16 W |
| *Hypsiboas punctatus A* | 193AF | EU201112 | X | French Guiana | Mana | New | 05 39 00 N 53 47 00 W |
| *Hypsiboas punctatus B* | MACN 37792 | AY549353 | X | Argentina | Chaco, Resistencia, Camino a Isla del Cerrito | Faivovich et al., 2004 | 27 14 01 S 58 37 00 W |
| *Hypsiboas raniceps A* | 15mc | AF467269 | X | French Guiana | Crique yiyi | Salducci et al., 2002 | 05 29 00 N 53 09 00 W |
| *Hypsiboas raniceps B* | MACN 37795 | AY843657 | X | Argentina | Santa Fe, Vera, Ea. 'Las Gamas' | Faivovich et al., 2005 | 29 28 01 S 60 12 08 W |
| *Hypsiboas semilineatus* | CFBH 5424 | AY843779 | | Brazil | Rio de Janeiro, Duque de Caxias | Faivovich et al., 2005 | 22 47 00 S 43 15 49 W |
| *Leptodactylus fuscus E* | USNM284551 | AY911279 | X | Brazil | Pernambuco | Camargo et al., 2005 | |
| *Leptodactylus gr. wagneri A* | 81mc | EU201128 | X | French Guiana | Montjoly | New | 04 55 00 N 52 16 00 W |
| *Leptodactylus gr. wagneri B* | 51bm | EU201125 | X | French Guiana | Kaw | New | 04 42 00 N 52 18 00 W |
| *Leptodactylus gr. wagneri C* | 170mc | EU201126 | X | French Guiana | Apatou | New | 05 10 00 N 54 20 00 W |
| *Leptodactylus gr. wagneri D* | 66bm | EU201127 | X | French Guiana | Mt Arawa | New | 02 48 59 N 53 21 59 W |
| *Leptodactylus gr. wagneri E* | 215mc | EU201129 | X | French Guiana | Apatou | New | 05 10 00 N 54 20 00 W |
| *Leptodactylus gr. wagneri F* | 78bm | EU201130 | X | French Guiana | Trinité | New | 04 35 00 N 53 21 00 W |
| *Leptodactylus gr. wagneri G* | 183af | EU201131 | X | Suriname | Road to Apura | New | 05 11 00 N 55 39 00 W |
| *Leptodactylus gr. wagneri H* | 155mc | EU201132 | X | French Guiana | Kaw | New | 04 42 00 N 52 18 00 W |
| *Leptodactylus gr. wagneri I* | 129af | EU201133 | X | Suriname | Brownsberg | New | 04 56 31 N 55 10 33 W |
| *Leptodactylus gr. wagneri J adenomera sp.* | AMNH-A 166312 | AY843561 | X | Guyana | Berebice River camp at ca 18 mi SWKwakwani, ca. 2 mi downriver from Kurundi River | Faivovich et al., 2005 | 05 05 06 N 58 14 14 W |
| *Leptodactylus knudseni A* | 52AF | EU201135 | X | French Guiana | Petit,saut | New | 05 04 00 N 53 03 00 W |
| *Leptodactylus knudseni B* | QCAZ13077 | AY947863 | X | Ecuador | Francisco de Orellana, Parque Nacional Yasuní, Napo | Heyer et al., 2005 | 00 26 56 S 77 00 55 W |
| *Leptodactylus leptodactyloides* | MZUSP 70969 | AY943236 | X | Brazil | Para, Serra de Kokoinhokren | de Sà et al., 2005 | 07 46 00 S 51 57 00 W |
| *Leptodactylus longirostris A* | 76bm | EU201119 | X | French Guiana | Mont arawa | New | 02 48 59 N 53 21 59 W |
| *Leptodactylus longirostris B* | 199mc | EU201120 | X | French Guiana | Grand santi | New | 04 20 00 N 54 15 00 W |
| *Leptodactylus longirostris B* | 103AF | EU201121 | X | Suriname | Road to Apura | New | 05 11 00 N 55 39 00 W |
| *Leptodactylus mystaceus A* | 134MC | EU201117 | X | French Guiana | Crique margot | New | 05 28 00 N 53 57 00 W |
| *Leptodactylus mystaceus B* | 115AF | EU201116 | X | Suriname | Brownsberg | New | 04 56 31 N 55 10 33 W |
| *Leptodactylus mystaceus C* | 73AF | EU201118 | X | French Guiana | St georges | New | 03 52 00 N 51 48 00 W |

| | | | | | | | |
|---|---|---|---|---|---|---|---|
| *Leptodactylus mystaceus D* | 167mc | EU201115 | X | French Guiana | Kaw | New | 04 42 00 N 52 18 00 W |
| *Leptodactylus mystaceus E* | 1126BPN | EU201114 | X | Guyana | Imbaimadai | New | 05 44 23 N 60 17 51 W |
| *Leptodactylus mystaceus F* | MZUSP 70371 | AY911286 | X | Brazil | Pará, Serra de Kukoinhokren | Camargo et al., 2005 | 07 46 00 S 51 57 00 W |
| *Leptodactylus ocellatus A* | 45mc | EU201124 | X | French Guiana | Guatemala | New | 05 09 00 N 52 38 00 W |
| *Leptodactylus ocellatus B* | MACN 38648 | AY843688 | X | Argentina | Buenos Aires, Escobar, Loma Verde,Ea. 'Los Cipreses' | Faivovich et al., 2005 | 35 16 56 S 58 25 18 W |
| *Leptodactylus ocellatus B* | KU 289191 | DQ158417 | X | Paraguay | Parque Nacional San Rafael | Pramuk, 2006 | 26 25 00 S 55 45 00 W |
| *Leptodactylus ocellatus C* | MZUSP 68993 | AY162395 | X | Brazil | SANTA CATARINA, Campeche | Nuin, 2002 | 27 40 17 S 48 28 33 W |
| *Leptodactylus pentadactylus A* | USNM 303466 | AY947868 | X | Brazil | Para, near Cachoeira do Espelho, ca. 50 km (airline) S of Altamira | Heyer et al., 2005 | 03 37 03 S 52 10 26 W |
| *Leptodactylus pentadactylus A* | MZUSP 70917 | AY947867 | O | Brazil | Pará, Serra de Kukoinhokren. | Heyer et al., 2005 | 07 46 00 S 51 57 00 W |
| *Leptodactylus pentadactylus A* | 181mc | EU201134 | X | French Guiana | Tibourou | New | 04 25 00 N 52 18 00 W |
| *Leptodactylus pentadactylus B* | MZUSP 70023 | AY947856 | X | Brazil | Pará, Aldeia A,Ukre | Heyer et al., 2005 | 03 37 23 S 49 20 32 W |
| *Leptodactylus pentadactylus B* | MZUSP 70075 | AY947857 | O | Brazil | Pará, Rio Vermelho | Heyer et al., 2005 | |
| *Leptodactylus pentadactylus C* | QCAZ 17056 | AY947864 | X | Ecuador | Esmeraldas, Alto Tambo. | Heyer et al., 2005 | 00 54 05 N 78 32 39 W |
| *Leptodactylus pentadactylus C* | QCAZ 19859 | AY947865 | O | Ecuador | Esmeraldas, Bosque Protector La Perla | Heyer et al., 2005 | 00 00 26 S 78 23 02 W |
| *Leptodactylus pentadactylus D* | C13095; MVZ 233238 | AY326017 | X | Costa Rica | Limon, Rio Penitencia, 2 mi N Tortuguero | Darst and Cannatella, 2003 | 10 35 31 N 83 31 40 W |
| *Leptodactylus pentadactylus D* | USNM 534219 | AY947862 | O | Honduras | Colon, Quebrada Machin | Heyer et al., 2005 | 15 33 18 N 85 42 45 W |
| *Leptodactylus pentadactylus D* | USNM 347153 | AY943238 | O | Panama | Bocas del Toro, Isla Popa | Heyer et al., 2005 | 09 09 09 N 82 07 57 W |
| *Leptodactylus pentadactylus D* | USNM 298079 | AY947866 | O | Panama | Bocas del Toro, Isla Popa | Heyer et al., 2005 | 09 09 09 N 82 07 57 W |
| *Leptodactylus rhodomystax A* | 213mc | EU201122 | X | French Guiana | Montagne des singes | New | 05 04 00 N 52 43 00 W |
| *Leptodactylus rhodomystax B* | 1248BPN | EU201123 | X | Guyana | Imbaimadai | New | 05 44 23 N 60 17 51 W |
| *Leptodactylus rhodomystax C* | MZUSP 70375 | AY947855 | X | Brazil | Pará, Serra de Kukoinhokren. | Heyer et al., 2005 | 07 46 00 S 51 57 00 W |
| *Leptodactylusfuscus A* | MZUSP67073 | AY911264 | X | Brazil | Roraima, Caracaranã, near Normandia | Camargo et al., 2005 | 03 50 00 N 59 47 00 W |
| *Leptodactylusfuscus A* | USNM291363 | AY911273 | X | French Guiana | Cayenne, Sinnamary | Camargo et al., 2005 | 05 23 00 N 52 57 00 W |
| *Leptodactylusfuscus A* | USNM497739 | AY911284 | O | Guyana | East Berbice, Region 10 cty. | Camargo et al., 2005 | 06 17 26 N 57 51 34 W |
| *Leptodactylusfuscus A* | AMNH A139088 | DQ283404 | X | Guyana | Southern Rupununi Savanna, Aishalton (onKubabawau Creek), 150 m | Frost et al., 2006 | 02 28 31 N 59 19 16 W |
| *Leptodactylusfuscus B* | USNM306189 | AY911269 | X | Panamá | Tocumen, Panamá City | Camargo et al., 2005 | 09 02 40 N 79 22 57 W |
| *Leptodactylusfuscus C* | USNM306067 | AY911265 | O | Tobago | Saint Paul, Roxborough | Camargo et al., 2005 | 11 15 00 N 60 35 00 W |
| *Leptodactylusfuscus C* | USNM306149 | AY911276 | O | Trinidad | Saint George, Arima, | Camargo et al., 2005 | 10 38 03 N 61 16 49 W |
| *Leptodactylusfuscus C* | USNM306123 | AY911270 | O | Trinidad | Manzanilla Mayaro Road, Nariva | Camargo et al., 2005 | 10 22 24 N 61 09 28 W |
| *Leptodactylusfuscus C* | USNM287012 | AY911272 | X | Trinidad | Saint Patrick, Icacos Point | Camargo et al., 2005 | 10 07 22 N 61 40 47 W |
| *Leptodactylusfuscus D* | MZUSP67039 | AY911277 | O | Brazil | Roraima, Bôa Vista | Camargo et al., 2005 | 02 44 14 N 60 42 40 W |
| *Leptodactylusfuscus D* | MZUSP76019 | AY911278 | X | Brazil | Roraima, Igarapé Cocal | Camargo et al., 2005 | 03 45 00 N 61 44 00 W |
| *Leptodactylusfuscus D* | | AY263226 | O | Venezuela | Canaima | Vences et al., 2003 | 06 13 58 N 62 50 59 W |
| *Leptodactylusfuscus E* | FML04789 | AY911274 | X | Argentina | Salta, Embarcación | Camargo et al., 2005 | 23 12 48 S 64 08 27 W |
| *Leptodactylusfuscus E* | USNMFS174020 | AY911267 | X | Bolivia | La Paz, Palos Blancos | Camargo et al., 2005 | 15 34 00 S 67 16 00 W |
| *Leptodactylusfuscus F* | USNM303149 | AY911266 | O | Brazil | São Paulo, Luiz Antonio | Camargo et al., 2005 | |
| *Leptodactylusfuscus F* | USNM303154 | AY911280 | O | Brazil | São Paulo, Luiz Antonio | Camargo et al., 2005 | |
| *Leptodactylusfuscus F* | USNM303155 | AY911281 | O | Brazil | São Paulo, Luiz Antonio | Camargo et al., 2005 | |
| *Leptodactylusfuscus F* | USNM303156 | AY911282 | O | Brazil | São Paulo, Luiz Antonio | Camargo et al., 2005 | |
| *Leptodactylusfuscus F* | USNM303157 | AY911283 | X | Brazil | São Paulo, Luiz Antonio | Camargo et al., 2005 | |
| *Leptodactylusfuscus G* | FML04788 | AY911271 | X | Argentina | Salta, Joaquín V. González | Camargo et al., 2005 | 24 48 33 S 65 21 47 W |
| *Leptodactylusfuscus H* | CBF02908 | AY911275 | X | Bolivia | Beni Biosphere Reserve | Camargo et al., 2005 | 13 24 01 S 64 33 20 W |
| *Leptodactylusfuscus I* | MZUSP66954 | AY911268 | X | Brazil | Pará, Serra de Kukoinhoken, Kenpore | Camargo et al., 2005 | 07 46 00 S 51 57 00 W |
| *Lithobates palmipes A* | KU 202896 | AY779211 | X | Ecuador | Napo, Misahualli, 600 m | Hillis and Wilcox, 2005 | 01 02 00 S 77 40 13 W |
| *Lithobates palmipes A* | 10mc | AF467266; AF467265 | X | French Guiana | Trois saut | Salducci et al., 2004 | 02 14 00 N 52 52 00 W |
| *Lithobates palmipes A* | AMNH A166454 | DQ283384 | X | Guyana | Magdalen's Creek camp, 300 yds NW bank of Konawaruk River ca. 25 mi (linear) WSW Mabura Hill, 400 ft | Frost et al., 2006 | 05 13 70 N 59 02 43 W |
| *Lithobates palmipes B* | AMNH A-118801 | AY779210 | X | Venezuela | Amazonas, Neblina Base Camp on Río Mawarinuma | Hillis and Wilcox, 2005 | 00 49 50 N 66 09 40 W |

| | | | | | | | |
|---|---|---|---|---|---|---|---|
| *Lithodytes lineatus A* | 55mc | EU201136 | X | French Guiana | Grand,Santi | New | 04 20 00 N 54 15 00 W |
| *Lithodytes lineatus A* | AMNH-A 166426 | AY843690 | X | Guyana | Berebice River camp at ca 18 mi (linear) SW Kwakwani (ca. 2 mi downriver from Kurundi River confluence) | Faivovich et al., 2005 | 05 05 06 N 58 14 14 W |
| *Lithodytes lineatus B* | N. Basso; USP 968438 | AY326012 | X | Brazil | Mato grosso, Apiacas | Darst and Cannatella, 2003 | 09 39 09 S 57 23 36 W |
| *Lithodytes lineatus B* | MZUSP 80874 | AY943241 | O | Brazil | Mato grosso, Apiacas | de Sà et al., 2005 | 09 39 09 S 57 23 36 W |
| *Lithodytes lineatus C* | LM 269 | U39988 | O | Peru | ? | Ruvinski and maxson, 1996 | |
| *Osteocephalus cabrerai* | JPC 13178; LSUMZ H-13720 | AY843705 | X | Brazil | Acre, Porto Walter | Faivovich et al., 2005 | 08 15 31 S 72 46 37 W |
| *Osteocephalus cabrerai* | 14mc | AF467267 | X | French Guiana | Kaw | Salducci et al., 2004 | 04 42 00 N 52 18 00 W |
| *Osteocephalus leprieurii A* | 141mc | EF376066 | X | French Guiana | Crique margot | Salducci et al., 2005 | 05 28 00 N 53 57 00 W |
| *Osteocephalus leprieurii B* | AMNH-A 1312546 | AY549361 | X | Venezuela | Amazonas, Neblina Base Camp on Rio Mawarinuma (=Rio Baria), 140 M | Faivovich et al., 2004 | 00 49 50 N 66 09 40 W |
| *Osteocephalus leprieurii B* | AMNH A-131254 | AY843707 | O | Venezuela | Amazonas, Neblina Base Camp on Rio Mawarinuma (=Rio Baria), 140 M | Faivovich et al., 2005 | 00 49 50 N 66 09 40 W |
| *Osteocephalus taurinus A* | 214mc | EF376067 | X | French Guiana | Saül | Salducci et al., 2005 | 03 37 32 N 53 12 26 W |
| *Osteocephalus taurinus B* | AMNH-A 131245 | AY843709 | X | Venezuela | Amazonas, Neblina Base Camp on Rio Mawarinuma (=Rio Baria), 140 M | Faivovich et al., 2005 | 00 49 50 N 66 09 40 W |
| *Osteocephalus taurinus C* | WED 55452; KU 205406 | AY326041 | X | Peru | Madre de Dios, Cusco Amazonico | Darst and Cannatella, 2003 | 11 39 00 S 70 33 35 W |
| *Phyllomedusa hypochondrialis* | 49mc | EF376079 | X | French Guiana | Grand,Santi | Salducci et al., 2005 | 04 20 00 N 54 15 00 W |
| *Phyllomedusa hypochondrialis* | MNH-A 141109; AMCC 101463 | AY843724 | X | Guyana | Dubulay Ranch on the Berbice River, 200ft | Faivovich et al., 2005 | 05 40 55 N 57 51 32 W |
| *Phyllomedusa hypochondrialis* | 109AF | EU201085 | X | Suriname | Brownsberg | New | 04 56 31 N 55 10 33 W |
| *Phyllomedusa tomopterna A* | 53mc | EF376077 | X | French Guiana | Kaw | Salducci et al., 2005 | 04 42 00 N 52 18 00 W |
| *Phyllomedusa tomopterna B* | 181AF | EU201086 | X | Suriname | Brownsberg | New | 04 56 31 N 55 10 33 W |
| *Phyllomedusa tomopterna C* | WED 55380; KU 205428 | AY326045 | X | Peru | Madre de Dios, Cusco Amazonico | Darst and Cannatella, 2003 | 11 39 00 S 70 33 35 W |
| *Phyllomedusa tomopterna D* | MJH 7076 | AY843728 | X | Peru | Huanuco, Rio Llullapichis, Panguana | Faivovich et al., 2005 | 09 23 02 S 75 52 57 W |
| *Phyllomedusa vaillanti* | 84bm | EU201087 | X | French Guiana | Petit,saut | New | 05 04 00 N 53 03 00 W |
| *Phyllomedusa vaillanti* | AMNH-A 1662888 | AY549363 | X | Guyana | Berbice River camp at ca.18 mi (linear)SW Kwakwan | Faivovich et al., 2004 | 05 05 06 N 58 14 14 W |
| *Pipa pipa A* | 11mc | EU201058 | X | French Guiana | Guatemala | New | 05 09 00 N 52 38 00 W |
| *Pipa pipa B* | KU 205801 | AY581621 | X | Peru | Madre de Dios, Cusco Amazonico 15 km E of Puerto Maldonado | Evans et al., 2004 | 12 34 48 S 69 03 51 W |
| *Pipa pipa C* | USNM 546385 | DQ283053 | X | Venezuela | Amazonas, Depto. Rio Negro, Neblina Base Camp on the Rio Baria, 140 m | Frost et al., 2006 | 00 49 50 N 66 09 40 W |
| *Ranitomeya amazonica* | DamaAema | AF482785 | | Peru | Almendras, Loreto, Peru | Symula et al., 2003 | 03 48 00 S 73 25 00 W |
| *Ranitomeya variabilis* | DvTY26b | AF412492 | | Peru | 26 km NE Tarapoto, San Martin, | Summers et al., 2001 | 06 21 37 S 76 24 45 W |
| *Ranitomeya ventrimaculata* | MNHN2000-0659 | AY263248 | O | French Guiana | Nouragues | Vences et al., 2003 | 04 05 00 N 52 41 00 W |
| *Ranitomeya ventrimaculata A* | 105mc | EU201084 | X | French Guiana | Tibourou | New | 04 25 00 N 52 18 00 W |
| *Ranitomeya ventrimaculata B* | 75bm | EU201083 | X | French Guiana | Pic coudreau | New | 03 18 02 N 52 56 77 W |
| *Ranitomeya ventrimaculata B* | BPN737 | DQ163076 | O | French Guiana | Kaw | Noonan and Wray, 2006 | 04 42 00 N 52 18 00 W |
| *Ranitomeya ventrimaculata B* | BPN741 | DQ163077 | O | French Guiana | Maripa | Noonan and Wray, 2006 | 03 48 52 N 51 53 06 W |
| *Ranitomeya ventrimaculata B* | BPN742 | DQ163078 | O | French Guiana | Maripa | Noonan and Wray, 2006 | 03 48 52 N 51 53 06 W |
| *Ranitomeya ventrimaculata B* | BPN744 | DQ163080 | O | French Guiana | Maripa | Noonan and Wray, 2006 | 03 48 52 N 51 53 06 W |
| *Ranitomeya ventrimaculata B* | BPN664 | DQ163075 | O | French Guiana | Pic Matecho | Noonan and Wray, 2006 | 03 45 00 N 53 02 00 W |
| *Ranitomeya ventrimaculata B* | BPN762 | DQ163081 | O | French Guiana | Saül | Noonan and Wray, 2006 | 03 37 32 N 53 12 26 W |
| *Ranitomeya ventrimaculata C* | JDL 24489 | DQ502266 | X | Colombia | Amazonas,Leticia,Km11(Leticia,Tarapaca) | Grant et al., 2006 | 04 10 06 S 69 54 41 W |
| *Ranitomeya ventrimaculata D* | OMNH 37440 | DQ502232 | O | Brazil | Amazonas, Castanho, ca. 40 km S Manaus,at km 12 on road to Autazes | Grant et al., 2006 | 03 37 10 S 59 86 78 W |
| *Ranitomeya ventrimaculata D* | DvenBraz1 | AF482797 | X | Brazil | Porto Walter, Acre, Brazil | Symula et al., 2003 | 08 15 31 S 72 46 37 W |
| *Ranitomeya ventrimaculata E* | OMNH 36666 | DQ502071 | X | Brazil | Amazonas, Rio Ituxi, Scheffer Madeireira | Grant et al., 2006 | 08 28 45 S 65 42 59 W |
| *Ranitomeya ventrimaculata E* | OMNH 36667 | DQ502072 | O | Brazil | Amazonas, Rio Ituxi, Scheffer Madeireira | Grant et al., 2006 | 08 28 45 S 65 42 59 W |
| *Ranitomeya ventrimaculata E* | LSUMZ H-3092 | DQ163079 | O | Brazil | Rio Ituxi, Amazonas | Noonan and Wray, 2006 | 08 28 45 S 65 42 59 W |
| *Ranitomeya ventrimaculata F* | ? | AF128619 | O | Ecuador | Pompeya | Clough and Summers, 2000 | 00 00 03 N 76 36 28 W |
| *Ranitomeya ventrimaculata F* | OMNH 34091 | DQ502069 | O | Ecuador | Sucumbios, Estacion Cientifica de Universidad Catolica near Reserva Faunistica Cuyabeno, 220m | Grant et al., 2006 | 00 00 00 S 76 10 00 W |
| *Ranitomeya ventrimaculata F* | QCAZ16566 | AY364570 | O | Ecuador | ? | Santos et al., 2003 | |
| *Ranitomeya ventrimaculata F* | DvenEcuador | AF482795 | X | Ecuador | Pompeya, Sucumbios | Symula et al., 2003 | 00 00 03 N 76 36 28 W |
| *Ranitomeya ventrimaculata G* | DvnNBon2 | AF412494 | X | Peru | Bonilla, San Martin, Peru | Symula et al., 2003 | 06 12 36 S 76 16 20 W |

| | | | | | | | |
|---|---|---|---|---|---|---|---|
| *Ranitomeya ventrimaculata H* | DvenNAPO | AF482796 | X | Peru | N. Bank Napo R., Loreto | Symula et al., 2003 | |
| *Ranitomeya ventrimaculata I* | Dvenorama | AF482791 | X | Peru | Allpahuayo, Loreto, Peru | Symula et al., 2003 | 03 52 52 S 73 26 02 W |
| *Ranitomeya ventrimaculata J* | OMNH 36062 | DQ502233 | O | Brazil | Acre, Porto Walter | Grant et al., 2006 | 08 15 31 S 72 46 37 W |
| *Ranitomeya ventrimaculata J* | MPEG 12394 | DQ502070 | X | Brazil | Acre, Porto Walter | Grant et al., 2006 | 08 15 31 S 72 46 37 W |
| *Ranitomeya ventrimaculata K* | DvnNBon1 | AF412493 | O | Peru | Bonilla, San Martin, Peru | Symula et al., 2003 | 07 02 46 S 76 47 00 W |
| *Ranitomeya ventrimaculata L* | DvenITYB8a2 | AF482799 | O | Peru | Nauta Road Iquitos, Loreto | Symula et al., 2003 | 04 34 14 S 73 46 00 W |
| *Rhaebo guttatus A* | 144mc | EF364307 | X | French Guiana | Crique margot | Fouquet et al., 2007 | 05 28 00 N 53 57 00 W |
| *Rhaebo guttatus A* | AMNH A141058 | DQ283375 | X | Guyana | Dubulay Ranch on the Berbice River, 200ft | Frost et al., 2006 | 05 40 55 N 57 51 32 W |
| *Rhaebo guttatus B* | LSUMZ 17418 | DQ158459 | X | Brazil | Rondonia | Pramuk, 2006 | |
| *Rhinella castaneotica* | LSUMZ 17429 | DQ158440 | | Brazil | Para, 100 km S Santarem | Pramuk, 2006 | 03 09 00 S 54 50 00 W |
| *Rhinella cf.margaritifera F* | QCAZ 13896 | DQ158471 | X | Ecuador | Cañar, Manta Real | Pramuk, 2006 | 02 30 44 S 79 04 56 W |
| *Rhinella cf.margaritifera F* | QCAZ 11597 | DQ158472 | O | Ecuador | Provincia Esmeraldas, Bosque Protector, 30 km from San Lorenzo by way of Ibarra | Pramuk, 2006 | 01 16 47 N 78 42 29 W |
| *Rhinella cf.margaritifera G* | QCAZ 10601 | DQ158470 | X | Ecuador | Francisco de Orellana, Parque Nacional Yasuní, Napo | Pramuk, 2006 | 00 26 56 S 77 00 55 W |
| *Rhinella cf.margaritifera I* | USNM 268828 | DQ158490 | X | Peru | Madre de Dios | Pramuk, 2006 | 11 39 00 S 70 33 35 W |
| *Rhinella cf.margaritifera J* | KU 215145 | DQ158491 | X | Peru | Madre de Dios | Pramuk, 2006 | 11 39 00 S 70 33 35 W |
| *Rhinella dapsilis* | QCAZ 3509 | DQ158448 | | Ecuador | Pichincha, Bosque Protector La Perla, 5 km E La Concordia | Pramuk, 2006 | 00 00 01 S 79 22 59 W |
| *Rhinella margaritifera A* | 204mc | EF364295 | X | French Guiana | Saül | Fouquet et al., 2007 | 03 37 32 N 53 12 26 W |
| *Rhinella margaritifera A 24 additional* | | | O | French Guiana | | Fouquet et al., 2007 | |
| *Rhinella margaritifera B* | 143PG | EF364302 | O | French Guiana | Kaw | Fouquet et al., 2007 | 04 42 00 N 52 18 00 W |
| *Rhinella margaritifera B* | 144PG | EF364302 | X | French Guiana | Kaw | Fouquet et al., 2009 | 04 42 00 N 52 18 00 W |
| *Rhinella margaritifera C* | 157mc | EF364303 | O | French Guiana | Tri jonction | Fouquet et al., 2007 | 02 20 00 N 54 36 00 W |
| *Rhinella margaritifera C* | 156mc | EF364303 | X | French Guiana | Tri jonction | Fouquet et al., 2011 | 02 20 00 N 54 36 00 W |
| *Rhinella margaritifera D* | 5mc | EF364304 | X | French Guiana | Cisame | Fouquet et al., 2007 | 04 11 00 N 52 22 00 W |
| *Rhinella margaritifera D 4 additional* | | | O | French Guiana | | Fouquet et al., 2007 | |
| *Rhinella margaritifera E* | 131bm | EF364287 | X | French Guiana | Mataroni | Fouquet et al., 2015 | 04 12 00 N 52 10 00 W |
| *Rhinella margaritifera E 17 additional* | | | O | French Guiana | | Fouquet et al., 2007 | |
| *Rhinella margaritifera G* | AGG172 (QCAZ) | AF375514 | O | Ecuador | Volcan Sumaco, Provincia Napo | Gluesenkamp, unpublished | 00 27 51 S 77 35 30 W |
| *Rhinella margaritifera H* | ZUEC (DCC 3393) | AY680262 | X | Brazil | Rio de Janeiro, Mage´, Campo de Escouteiras, Santo Aleixo | Pauly et al., 2004 | 22 33 54 S 43 04 09 W |
| *Scinax boesemani A* | 147mc | EF217501 | X | French Guiana | Guatemala | Fouquet et al., 2007 | 05 09 00 N 52 38 00 W |
| *Scinax boesemani A 7 additional* | | | O | French Guiana | | Fouquet et al., 2007 | |
| *Scinax boesemani B* | 932BPN | EU201090 | X | Suriname | Road to Apura | New | 05 11 00 N 55 37 00 W |
| *Scinax boesemani C* | 1022BPN | EU201089 | X | Suriname | Sipilawini | New | 02 02 00 N 56 07 00 W |
| *Scinax boesemani D* | 198mc | EF217503 | X | French Guiana | Grand Santi | Fouquet et al., 2007 | 04 20 00 N 54 15 00 W |
| *Scinax boesemani D* | 39mc | EF376072 | O | French Guiana | Grand Santi | Salducci et al., 2005 | 04 20 00 N 54 15 00 W |
| *Scinax boesemani E* | 1169BPN | EU201088 | X | Guyana | Mazaruni,Potaro Imbaimadai | New | 05 44 23 N 60 17 51 W |
| *Scinax cf. cruentommus A* | 8mc | AF467263 | X | French Guiana | Kaw | Salducci et al., 2004 | 04 42 00 N 52 18 00 W |
| *Scinax cf. cruentommus A 20 additional* | | | O | French Guiana | | Fouquet et al., 2007 | |
| *Scinax cf. cruentommus B* | 324mc | EU201093 | X | French Guiana | Antecum,Pata | New | 03 19 00 N 54 04 00 W |
| *Scinax fuscovarius* | MACN 38647 | AY843758 | X | Argentina | Misiones, Guarani, San Vicente, Campo Anexo INTA 'Cuartel Rio Victoria | Faivovich et al., 2005 | 26 56 00 S 54 24 00 W |
| *Scinax nasicus* | MACN 38650 | AY843759 | X | Argentina | Buenos Aires, Baradero, Estancia 'El Retonio' | Faivovich et al., 2005 | 33 48 44 S 59 30 25 W |
| *Scinax nebulosus B* | 394BPN | EU201094 | X | Brazil | State of Pernambuco, Timbaúba, Engenho Água Azul | New | 06 38 05 S 37 54 02 W |
| *Scinax nebulosus A* | 24mc | AF467262 | X | French Guiana | Régina,St Georges Road | Salducci et al., 2004 | 04 03 00 N 52 01 00 W |
| *Scinax nebulosus A 2 additional* | | | O | French Guiana | | Fouquet et al., 2007 | |
| *Scinax nebulosus D* | AA900 | EU201095 | X | Guyana | Barima,Waini Mabaruma | New | 08 12 00 N 59 46 48 W |
| *Scinax nebulosus C* | CE581 | EU201096 | X | Brazil | Ceará State, Ibiapina, Sítio Pimentas, Vivenda Santa Rosa | New | 03 55 00 S 40 53 00 W |
| *Scinax ruber A* | 74mc | EF217478 | X | French Guiana | Montagne d'argent | Fouquet et al., 2007 | 04 23 00 N 51 42 00 W |
| *Scinax ruber A 15 additional* | | | O | French Guiana | | Fouquet et al., 2007 | |

| | | | | | | | |
|---|---|---|---|---|---|---|---|
| *Scinax ruber B* | 151mc | EF217481 | X | French Guiana | Guatemala | Fouquet et al., 2007 | 05 09 00 N 52 38 00 W |
| *Scinax ruber B 10 additional* | | | O | French Guiana | | Fouquet et al., 2007 | |
| *Scinax ruber C* | WED 56265; KU 207622 | AY326034 | X | Peru | Madre de Dios, Cusco Amazonico | Darst and cannatella, 2003 | 11 39 00 S 70 33 35 W |
| *Scinax ruber D* | 40mc | EF376073 | O | French Guiana | | Fouquet et al., 2007 | |
| *Scinax ruber D* | 178mc | EF376073 | X | French Guiana | Ouanary | Salducci et al., 2005 | 04 15 00 N 51 40 00 W |
| *Scinax ruber D* | 164AF | EU201092 | X | Suriname | Brownsberg | New | 04 56 31 N 55 10 33 W |
| *Scinax ruber D 3 additional* | | | O | French Guiana | | Fouquet et al., 2007 | |
| *Scinax ruber E* | IWK 109 | AY549365 | X | Guyana | Iwokrama, Muri Scrub camp | Faivovich et al., 2004 | 04 40 17 N 58 41 06 W |
| *Scinax ruber F* | 76mc | EF217490 | X | French Guiana | Mont Ravel | Fouquet et al., 2007 | 04 54 42 N 52 15 39 W |
| *Scinax ruber F 9 additional* | | | O | French Guiana | | Fouquet et al., 2007 | |
| *Scinax ruber G* | 360mc | EU201091 | X | French Guiana | Mont Ravel | New | 04 54 42 N 52 15 39 W |
| *Scinax ruber H* | QCAZ25275 | EF217487 | X | Ecuador | AUCA14road,parroquia Dayuma,canton coca,Orellana | Fouquet et al., 2007 | 01 04 03 S 77 36 55 W |
| *Scinax ruber H 3 additional* | | | O | Ecuador | | Fouquet et al., 2007 | |
| *Scinax x-signatus* | 144bm | EF217479 | X | French Guiana | Kaw | Fouquet et al., 2007 | 04 42 00 N 52 18 00 W |
| *Sphaenorhynchus lacteus* | 85mc | EF217515 | X | French Guiana | Kaw | Fouquet et al., 2007 | 04 42 00 N 52 18 00 W |
| *Sphaenorhynchus lacteus* | USNM 268930 | AY549367 | X | Peru | Madre de Dios, Tambopata Reserve | Faivovich et al., 2004 | 14 13 25 S 69 10 36 W |
| *Trachycephalus hadroceps* | MNHN 2001.0814 | AY843717 | | French Guiana | Kaw | Faivovich et al., 2005 | 04 42 00 N 52 18 00 W |
| *Trachycephalus resinifictrix* | AMNH-A 131201; AMCC 101463 | AY843719 | | Venezuela | Amazonas, Neblina Base Camp on Rio Mawarinuma, 140 m | Faivovich et al., 2005 | 00 49 50 N 66 09 40 W |
| *Trachycephalus venulosus A* | 163mc | EF376069 | X | French Guiana | Guatemala | Salducci et al., 2005 | 05 09 00 N 52 38 00 W |
| *Trachycephalus venulosus A* | AMNH-A 1411427 | AY549362 | X | Guyana | Dubulay Ranch on the Berbice River, 200ft | Faivovich et al., 2004 | 05 40 55 N 57 51 32 W |
| *Trachycephalus venulosus B* | DCC 3069; TNHC 62490 | AY326048 | X | Ecuador | ? | Darst and cannatella, 2003 | |
| *Trachycephalus venulosus C* | ? | AY364350 | O | ? | ? | Biju and Bossuyt, 2003 | |
| *Trachycephalus venulosus C* | VUB 0987 | AY364371 | X | ? | ? | Biju and Bossuyt, 2003 | |
