# Supplementary figures and images for: Underestimation of Species Richness in Neotropical Frogs Revealed by mtDNA Analyses
Source: PLoS One. 2007 Oct 31;2(10):e1109. doi: 10.1371/journal.pone.0001109 (PMC2040503; doi:10.1371/journal.pone.0001109)

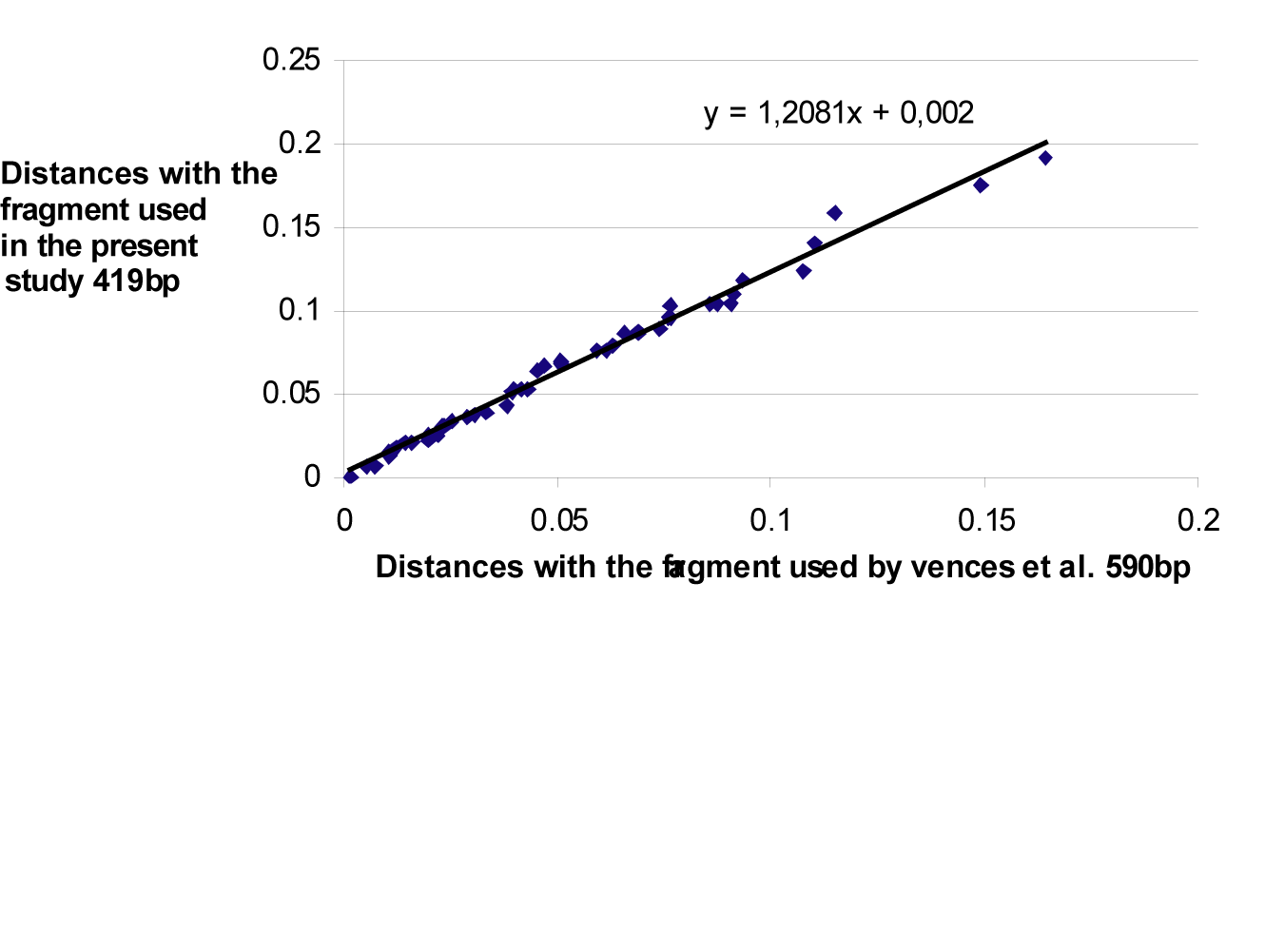

Supplement: Figure S1 — Distribution of the pairwise distances between the Hylinae sister species from Faivovich et al. (2005) with two sizes of the same 16S rDNA fragment: One with 590bp corresponding to the fragment used by Vences (2005) and one with 419bp for the present study. (3.99 MB TIF) [file pone.0001109.s003.tif]

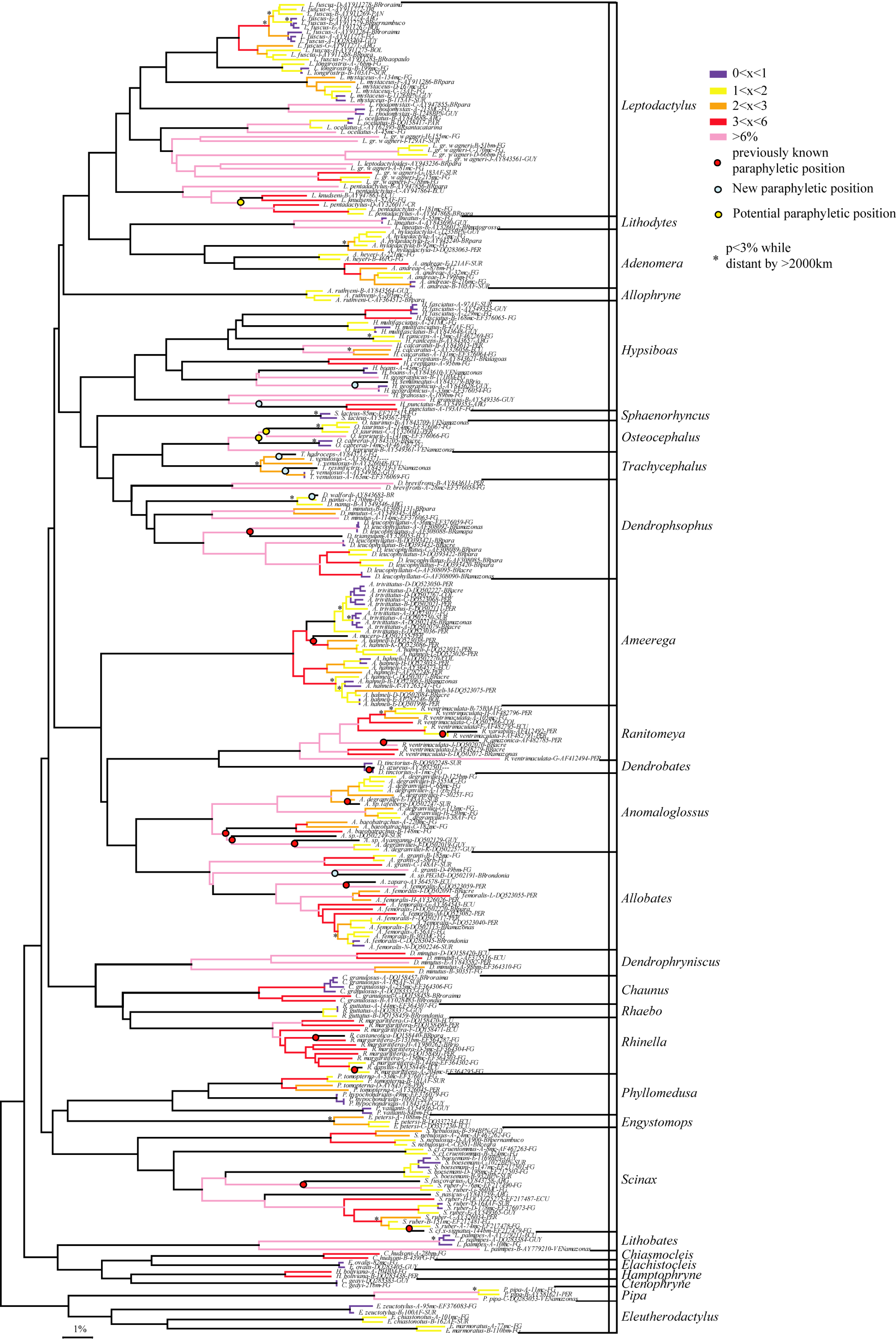

Supplement: Figure S2 — Tree from Figure 1 with sample labels and geographical indications: FG = French Guiana; SUR = Suriname; GUY = Guyana; VEN = Venezuela; BR = Brazil; COL = Colombia; PAN = Panama; CR = Costa Rica; ECU = Ecuador; PER = Peru; BOL = Bolivia; PAR = Paraguay; ARG = Argentina. (6.89 MB TIF) [file pone.0001109.s004.tif]

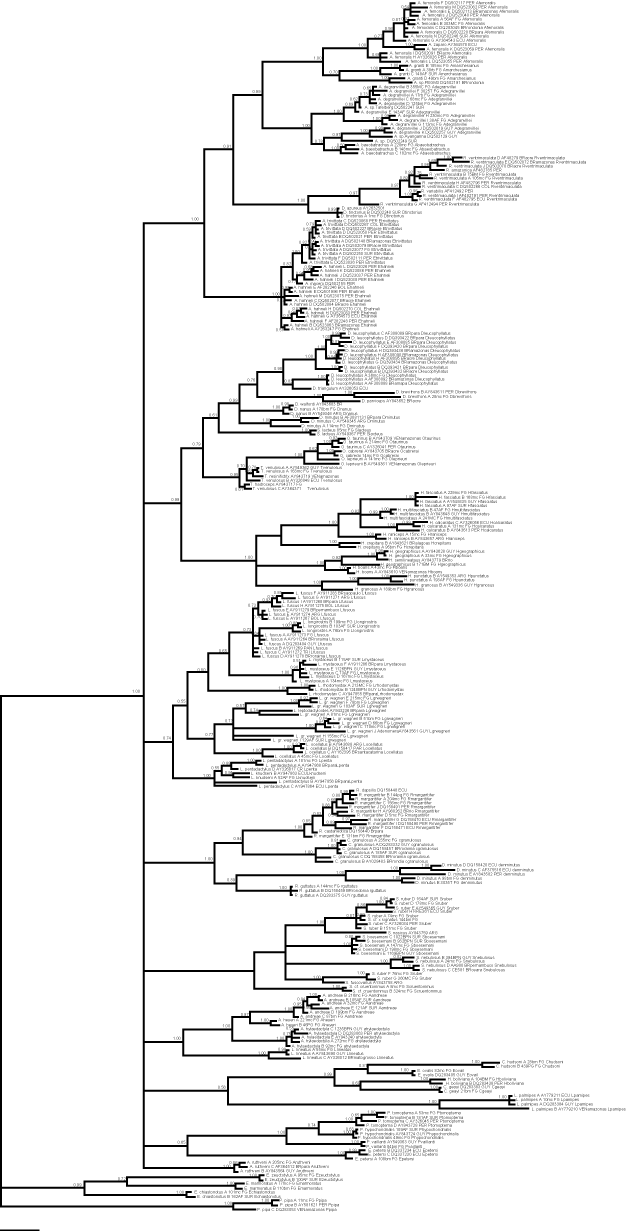

Supplement: Figure S3 — Consensus tree derived from Bayesian analysis of the data (2.31 MB TIF) [file pone.0001109.s005.tif]
